# Social anxiety changes the way we move—A social approach-avoidance task in a virtual reality CAVE system

**DOI:** 10.1371/journal.pone.0226805

**Published:** 2019-12-23

**Authors:** Bastian Lange, Paul Pauli

**Affiliations:** 1 Department of Psychology, Biological Psychology, Clinical Psychology, and Psychotherapy, University of Würzburg, Würzburg, Germany; 2 Center of Mental Health, Medical Faculty, University of Würzburg, Germany; Universitatsklinikum Tubingen, GERMANY

## Abstract

Investigating approach-avoidance behavior regarding affective stimuli is important in broadening the understanding of one of the most common psychiatric disorders, social anxiety disorder. Many studies in this field rely on approach-avoidance tasks, which mainly assess hand movements, or interpersonal distance measures, which return inconsistent results and lack ecological validity. Therefore, the present study introduces a virtual reality task, looking at avoidance parameters (movement time and speed, distance to social stimulus, gaze behavior) during whole-body movements. These complex movements represent the most ecologically valid form of approach and avoidance behavior. These are at the core of complex and natural social behavior. With this newly developed task, the present study examined whether high socially anxious individuals differ in avoidance behavior when bypassing another person, here virtual humans with neutral and angry facial expressions. Results showed that virtual bystanders displaying angry facial expressions were generally avoided by all participants. In addition, high socially anxious participants generally displayed enhanced avoidance behavior towards virtual people, but no specifically exaggerated avoidance behavior towards virtual people with a negative facial expression. The newly developed virtual reality task proved to be an ecological valid tool for research on complex approach-avoidance behavior in social situations. The first results revealed that whole body approach-avoidance behavior relative to passive bystanders is modulated by their emotional facial expressions and that social anxiety generally amplifies such avoidance.

## Introduction

According to the motivational priming hypothesis [1], appetitive and aversive cues prime the organism and pre-activate the appetitive or the defensive motivational system, respectively, and thereby prepare for approaching positive stimuli and avoiding negative stimuli.

In the laboratory, approach and avoidance behaviors to affective stimuli have been extensively studied using approach-avoidance tasks (AATs) which mainly assess hand movements [2]. For example, in a classical study by Chen and Bargh [3], participants had to respond to words with positive (e.g., “puppy”) or negative (e.g., “cockroach”) valence by either pushing or pulling a lever. Notably, stimuli with a positive valence facilitated arm flexions which represent approach behavior, while negative stimuli facilitated arm extension representing avoidance behavior. This kind of task has been replicated many times with varying set-ups, for example using controllers instead of levers and affective pictures instead of words [3–8]. However, one limitation of these tasks is that they typically involve arm movements restricted to one axis only, i.e. they allow no inference about approach or avoidance behavior of the person, i.e. whole-body movements. The AAT was also used to examine the effects of faces, which are assumed to be salient social and emotional cues, on approach-avoidance behaviors [9]. Several studies using emotional facial expressions replicated the classical AAT findings [10–12], for example an angry facial expression was found to facilitate avoidance-related behaviors, i.e. arm extensions [10]. Moreover, a meta-analysis concluded that approach-avoidance effects for faces are significantly larger compared with affective words or pictorial stimuli [13]. The AAT with facial stimuli has further been used to study approach-avoidance tendencies in social anxiety [14–16].

Social anxiety disorder (SAD) is one of the most common psychiatric disorders, with a lifetime prevalence of up to 12% in Western societies [17–20]. This disorder is characterized by an excessive fear of being evaluated by others [21, 22]. The current DSM-5 defines SAD as having a persistent fear of social situations and performance situations in which people with SAD are exposed to unfamiliar people or to the possibility of being scrutinized by others [22]. Fundamental research has identified cognitive factors as crucial components in the development of social fears [23–27]. The development of SAD is supported by negative basic beliefs, which are formed through bad experiences during childhood and adolescence. Next to the development of SAD, its maintenance is a crucial aspect for understanding and treating the disorder. Here the two leading cognitive models from Clark and Wells [24] and Rapee and Heimberg [27], state that avoidance behavior plays a significant role. In SAD avoidance behavior contains not only the apparent act of not entering social situations at all, but also to avoid the negative evaluation of others [24, 27, 28], for example avoidance of eye contact or avoidance of close body contact. In other words, people suffering SAD perceive social interactions as threatening and therefore avoid them. Due to its importance in maintaining the disorder, it seems crucial to investigate how and to what extent avoidance of social stimuli is manifested in patients suffering SAD.

For example, one study using the AAT compared participants high in social anxiety with non-anxious controls and revealed that high social anxiety was associated with an increased avoidance tendency, i.e. pushing a joystick away, related to emotional facial expressions compared to neutral faces [14]. However, in everyday life it is difficult to completely avoid social situations and consequently high socially anxious people also display more subtle avoidance behavior [29], i.e. avoidance of eye contact.

In everyday life and in more natural situations approach and avoidance behaviors are not simply restricted to the flexion or extension of the arm. In its most basic sense, approach and avoidance means to decrease or to increase the distance between oneself and an environmental cue, respectively. Following this logic, whole-body movements represent the most direct and ecologically valid forms of approach and avoidance behavior [30]. Supportively, Stins and colleagues [12] modified the AAT and asked participants to step towards or away from a monitor showing emotional facial expressions, and they observed slower approach to angry faces compared to happy faces.

Another way to investigate more complex and natural social behavior is to study interpersonal distance, which is the distance people keep between themselves and others in an automatic manner. Commonly, in order to examine moderators of personal distance participants are asked to approach another person or are approached by another person, who is the confederate of the experimenter, and then have to indicate when the personal distance starts to feel uncomfortable [31–33]. However, results are quite inconsistent as the review from Hayduk [34] already highlighted. A more recent article states that confederates act differently depending on their interaction partners [35], which in turn influences how participants behave [36]. In addition, it has been argued that using confederates lacks ecological validity, and because of incomplete control of their behavior and their facial expressions the obtained measures of personal distance are unreliable or inaccurate [33]. Thus, research on personal distance has been scarce.

The study of Stins *et al*. [12] and the research on personal distance are steps in the direction of increasing ecologically validity and examining more natural and whole body approach and avoidance behaviors in social situations. Further methodological improvements allowing high ecological validity and perfect experimental control leading to high internal validity may be possible by using virtual reality (VR) [37–39]. Virtual persons, i.e. agents (controlled by computers) or avatars (controlled by humans), can be used to reenact ecological valid social encounters under strict experimental control. Moreover, important factors such as gender of the interaction partner, gaze direction, movement and facial expressions can be manipulated systematically [38, 39]. Another advantage of VR is that it allows reliable and implicit registration of behavior by means of tracking head, body and eye movements.

Over the last years several studies successfully applied VR to study approach-avoidance behavior and grossly replicated the above described results [29, 37, 40–46]. Thus, agents with angry facial expressions were physically more avoided than agents with sad or neutral facial expressions [44]. Interestingly, people with social anxiety disorder showed a slower approach towards virtual persons and kept larger distances from them [46]. In line, Wieser *et al*. [29] found that, when approached by agents displaying a direct gaze, women high in social anxiety avoided gaze contact at greater distances and showed stronger avoidance behavior (i.e., backward head movements). In the VR studies described, above the participants’ task was either to move towards an agent or they were approached by the agent. Studies so far mainly assessed behavior explicitly related to an upcoming direct social interaction. A research question that remains is how behavior, not explicitly related to the interaction with others, is affected by social anxiety. This seems crucial, because as mentioned before patients maintain SAD not just by showing avoidance behaviors when directly engaging in social interactions, but also in social situations in general. In addition, it seems important to allow participants natural body movements which in previous VR studies was mostly restricted because participants had to wear a head mounted display (HMD). A HMD restricts the view of the own body and/or allows “joystick” movements only, i.e. participants do not move physically but receive simulated visual feedback from body movements controlled by joystick movements.

The present study addresses these issues and therefore differs from previous research in two important aspects. Firstly, it examines the implicit and subtle effects of social factors on behavior with no explicit relation to other persons. For example, we predict that approaching a specific location (e.g., a shop) implicitly leads to different behavior if we have to pass by a bystander or not and if the bystander shows a friendly or unfriendly facial expression. To examine this systematically, the present study introduces a VR task during which participants must pass by a virtual agent which varies in the facial expression on their way to a specific target location. The agent is irrelevant for the main task and any action or attention towards the agent therefore is voluntary. This enables this research to study implicit social approach-avoidance behavior, rather than behavior in direct explicit social interactions. Secondly, this study differs from the above described VR studies in the form of the used equipment. By using projection methods within a 5-sided CAVE system, participants could move around naturally and fairly unrestricted from any VR equipment. The CAVE allows the participants to walk physically as in natural situations and simultaneously seeing their own body. Both factors are considered as important for an ecologically valid assessment of behavior in social situations. Three hypotheses are tested. First, we expected that all participants exhibit enhanced avoidance behavior, for instance, greater distance and less eye contact, when bypassing an agent with a negative compared to an agent with a neutral facial expression. Secondly, we predicted that social anxiety modulates such avoidance behavior with high socially anxious participants showing generally more avoidance behavior towards all agents. Finally, we hypothesised that the avoidance behavior of socially anxious participants is specifically exaggerated towards an agent with a negative facial expression.

## Materials and methods

### Participants

Participants were selected by means of an online screening on the basis of a social anxiety screening questionnaire which was previously used successfully for recruiting socially anxious participants [47–49]. Informed consent was obtained by all participants before the online screening. The questionnaire consists of five 5-point Likert scale items, which are based on the criteria for social phobia from the Diagnostic and Statistical Manual of Mental Disorders [21]. An average score of 3.2 or higher was used as cut-off value for the selection of high socially anxious (HSA) participants, based on the study from Reutter *et al*. [48], where participants in this range made up the upper 19.26% of all individuals screened. For the control group, participants with scores between 1.6 and 2.2 were selected and matched to the HSA participants according to gender, age and education. Besides that, all participants gave demographic information and completed two other questionnaires (a fear of heights and a personality questionnaire) which were added to make it less apparent that the screening was solely conducted to select high socially anxious people, thereby minimizing the participation bias.

For the present study 376 people completed the online questionnaire, 287 females and 89 males with a mean age of 26.31 years (*SD* = 8.51). The mean social anxiety screening score of all participants was 2.31 (*SD* = 0.99), with 89 belonging to the HSA group (=> 3.2), 104 to the control group (=> 1.6 and =<2.2); the remaining individuals had either to low scores (< 1.6) or fell between the HSA and control criteria (> 2.2 and < 3.2). In our screening sample a score above 3.2 represented the upper 23.67%, thereby confirming Reutter *et al*. [48], that the cut-off represents the upper 20% of individuals screened online. The control range between 1.6 and 2.2 was also chosen on the basis screening data of Reutter *et al*. [48] with the intention to select participants who do not belong to the lowest 20% and were not on the border of being high socially anxious, thus we intended to select control participants who were neither low, nor high in social anxiety.

From the 376 people completing the online screening, 52 individuals participated in the study. However, one female participant from the HSA group had to be excluded due to technical problems with the tracking system, and therefore her matched control was excluded as well. This left 50 participants (25 HSA, 25 matched controls) for the statistical analysis. All participants signed the informed consent, reported normal or corrected-to-normal vision and received 10 € for their participation. The study was approved by the ethics committee of the University of Würzburg and was in accordance with the Helsinki declaration. Participants were told, that the study investigates movement in VR. The real purpose of the study was not revealed to prevent influences on the participants’ behavior. At the end of the experiment, the subjects were verbally informed that this information was withheld. At the end of the study, the participants were given a full explanation on request.

During the study, participants answered several questionnaires. The state part of the State-Trait Anxiety Inventory (STAI) [50] and the Self-Assessment Manikin questionnaire (SAM) [51] were used to assess the current state of the participant before and after the experiment, as best estimates of anxiety and emotionality during the VR task. All other questionnaires were answered at the end of the study, including a sociodemographic questionnaire, the trait part of the STAI, the Igroup Presence Questionnaire (IPQ) [52], and the Simulator Sickness Questionnaire (SSQ) [53]. Finally, the Social Phobia and Anxiety Inventory (SPAI) [54] and again the above described social anxiety screening questionnaire were filled in. Test-retest reliability of the screening questionnaire resulted in a correlation of 0.93. As can be seen in Table 1, the two groups showed the same scores for most of the questionnaires’ scores. However, as expected HSA participants had higher scores on social anxiety questionnaires (SPAI, pre-screen questionnaire), as well as higher trait anxiety (trait part of the STAI). Furthermore, groups differed in levels of nausea after completing the experiment (SSQ-nausea). It may be that taking part in the study is especially exciting for HSA participants. This increased excitement may have led to higher nausea levels. However, to rule out confounding effects of the group difference in levels of nausea on the later analysis, we calculated the correlations between the Simulator Sickness Questionnaire (SSQ) nausea scores and the approach and avoidance parameters. The analysis did not return any correlations exceeding a small correlation effect (r >= .30). Therefore, any influence of the group difference in levels of nausea on the later analysis of the approach and avoidance parameters can be ruled out.

**Table 1:** Group characteristics.

	HSA		control				
Variable	*M*	*SD*	*M*	*SD*	t(48)	p value	cohens d
Age	23.9	3.5	24.1	3.3	- 0.20	.840	-0.06
Social Anxiety Screening (online)	3.7	0.4	2.0	0.3	16.22	.001	4.50
Social Anxiety Screening (laboratory)	3.7	0.5	1.9	0.5	12.49	.001	3.46
SPAI	3.0	0.9	1.7	0.7	5.52	.001	1.53
STAI (trait)	46.7	9.8	35.7	7	5.93	.001	1.68
SSQ (nausea)	24.2	21.6	12.5	13.4	2.35	.023	0.65
SSQ (oculomotor)	26.8	17.2	23.6	20.5	0.61	.544	0.17
SSQ (disorientation)	25.7	31.7	19.3	29.0	0.76	.449	0.21
IPQ (spatial presence)	4.1	1.0	3.9	1.1	0.56	.577	0.16
IPQ (involvement)	3.7	1.2	3.2	1.3	1.37	.178	0.38
IPQ (experienced realism)	2.8	1.1	2.8	1.0	0.00	.999	0.01
	female	male	female	male			
Gender	19	6	19	16			

HSA, high socially anxious; SPAI, Social Phobia and Anxiety Inventory; STAI, State–Trait Anxiety Inventory; SSQ, Simulator Sickness Questionnaire; IPQ, Igroup Presence Questionnaire.

Repeated measures ANOVAs with the factors time (pre/post experiment) and group (HSA/control) on the participants emotional state before and after the experiment revealed no effects involving the factor group and only marginally significant effects of time for state anxiety and valence ratings suggesting a reduction in state anxiety and a deterioration of reported valence, independent of group (see Table 2).

**Table 2:** State change over time.

	Pre		Post				
Variable	*M*	*SD*	*M*	*SD*	*F*(1,48)	*p* value	$\eta_{p}^{2}$
STAI (state)	37.1	7.4	35.1	7.1	3.59	.064	0.07
SAM (arousal)	6.08	1.6	6.26	1.8	0.63	.432	0.01
SAM (valence)	2.92	1.4	2.58	1.4	2.90	.095	0.06
SAM (control)	6.36	1.6	6.70	1.6	2.78	.102	0.05

Pre, scores assessed at the beginning of the experiment; Post, scores assessed at the end of the experiment; STAI, State–Trait Anxiety Inventory; SAM, Self-Assessment Manikin questionnaire.

### Virtual reality apparatus

For immersion of participants in the VR, a 3D-multisensory CAVE laboratory was used consisting of a 5-sided Cave Automatic Virtual Environment (by BARCO, Kuurne, Belgium). With six projectors the VR scene was projected on the four walls and the floor (length x width x height: 4 x 3 x 3 m). Four projection surfaces had a resolution of 1920 x 1200 pixel and one had a higher resolution of 2016 x 1486, due to an additional projector. To induce three-dimensional depth, stereoscopic images were created using two computers for each surface and passive interference-filtering-glasses (Infitec Premium, Infite, Ulm, Germany). Audio stimuli were presented with a 7.1 surround system. An active infrared LED tracking system with four cameras (PhaseSpace Impulse, PhaseSpace Inc., San Leandro, CA, USA) was employed in order to capture movement and orientation data. Data were recorded with a sampling rate of 60 Hz. The virtual environment was generated by a Source SDK (Valve Corporation, Bellevue, Washington, USA) based modification (VrSessionMod 0.6). Experimental control and data recording were established using the VR-software CyberSession (CS-Research 5.6, VTplus GmbH, Würzburg, Germany; see www.cybersession.info for details). The VR-software was executed on an additional computer, which was also running the rendering control unit.

### Virtual reality environment

The virtual environment consisted of a room of the CAVE’s physical dimension (length x width x height: 4 x 3 x 3 m). Participants could move around freely (without the necessity of additional equipment, e.g. a gamepad) and were equipped with 3D glasses, a clip-on microphone for communication with the experimenter and a handheld controller with buttons for giving responses. The position of the 3D glasses was tracked with active infrared LED tracking to adapt the 3D images to the position and orientation of the head. Thereby height differences between participants were automatically adjusted. Each of the four walls had a reddish brick stone pattern, which was chosen for two reasons. Firstly, it enhanced the visibility of the agents. Second, the background gave the feeling of being in a backyard ally and thereby enhanced the ecological validity. The floor had a white marble pattern. Temporary virtual elements marked the start position (red footsteps) and the target position (green circle). Dependent on the experimental condition participants had to pass a virtual agent to reach the target position. The virtual agent stood in one position showing random idle behavior and again depending on the experimental condition displayed different facial expressions and followed the participant with gaze and body orientation. In total three different male agents were used. The facial expressions were designed using the SDK tool faceposer, which is based on the Facial Action Coding System [55, 56].

### Procedure

After arriving at the laboratory and answering the above described questionnaires and signing the informed consent, participants were equipped with the necessary tools for VR immersion and positioned in the CAVE. Participants could interact with the experimenter via microphone. In addition, the experimenter could monitor the participant inside the CAVE by means of a video screen. Before participants were immersed in VR, they were instructed that they could stop the experiment at any moment, without giving any reason. Instructions of the experiment were pre-recorded and played back via loudspeakers. Participants could navigate through the instructions via button presses on the controller. After instructions and before the main session started, participants had the opportunity to ask questions. During the instruction phase participants executed four consecutive test trials, to familiarize with the task.

**Fig 1:**
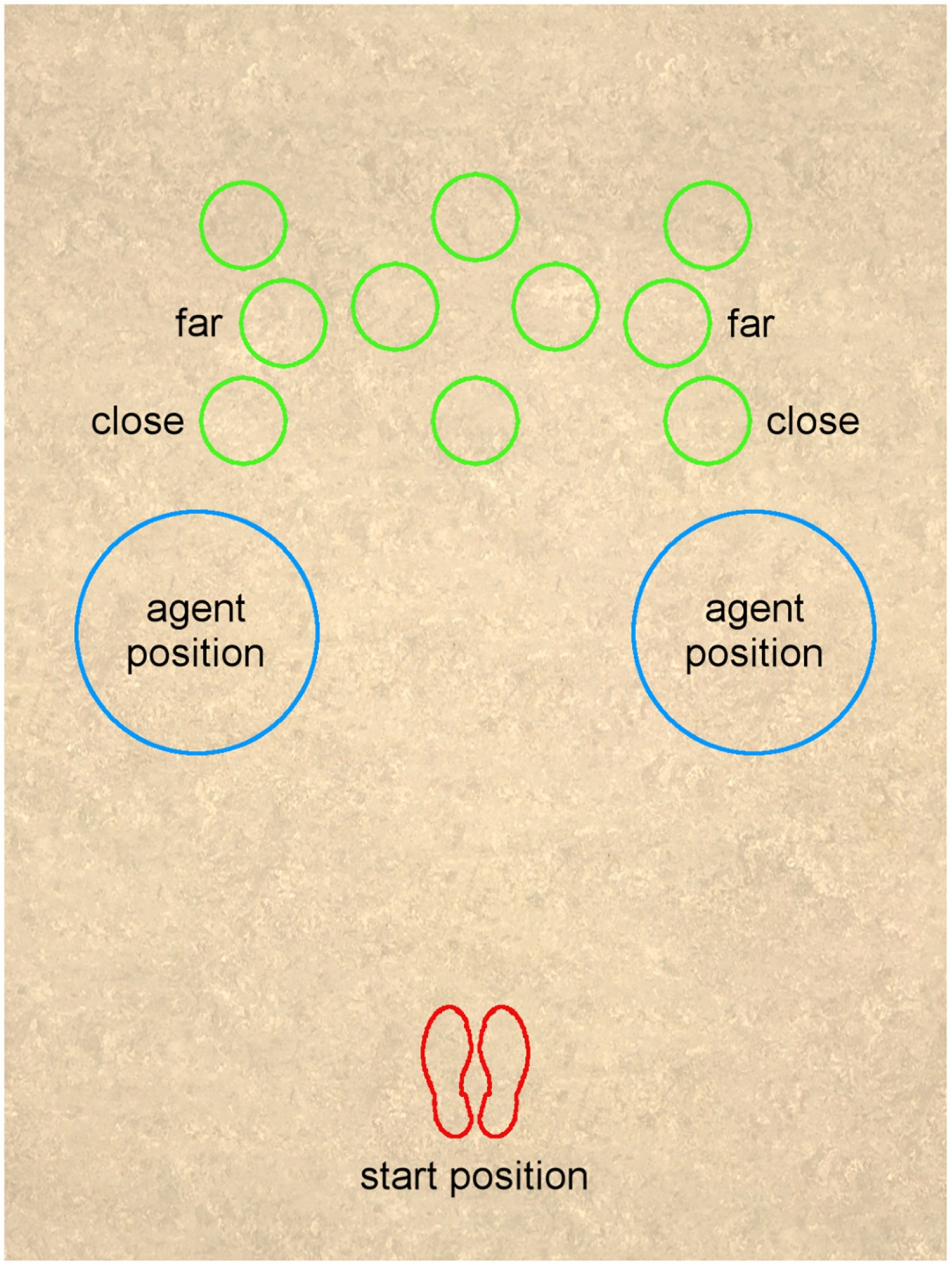
Projection on CAVE floor. Floor of the CAVE from above showing outlines for the start position (red) and all possible target positions (green), as well as the possible agent positions (blue) and the four “targets of interest” positions, labeled as close and far.

At the beginning of each trial the participants were asked to position themselves on the start location, indicated with red footprints (see Fig 1). When ready to start, they pressed a button on the controller which caused an agent to appear at one of two locations (see Fig 1). The agent’s dynamic facial expression was either neutral or angry throughout the trial. Furthermore, the agent’s whole-body posture always faced towards the participant and followed the participant with its eyes fixating the participant’s 3D glasses thereby giving the impression of looking at the participant’s eyes. When an agent appeared, participants had to name its hair color, thereby it was assured that participants recognized the agent’s face. Afterwards, the target location mark (filled green circle with 27.43 cm (10.8 in) diameter) blinked up on the virtual floor for 100 ms. For the main task, participants were instructed to move to the target position as quickly and accurately as possible. When they reached the position, they pressed a button to end the trial. After 500 ms the start location was visible again, so that participants could start the next trial. It is important to note that although participants have to name the hair color of the agent, the main task of going to the target location by bypassing the agent does not require any explicit social interaction.

The target could be presented in one out of ten target locations (see Fig 1 for all possible locations). However, out of those ten locations, only four were of interest (close and far labeled target positions in Fig 1). The remaining six target locations were used for dispersion and scattered throughout the room. From the total number of 108 trials, 36 were dispersion trials with one of the six irrelevant target locations (see Table 3). In 12 of these dispersion trials no agent was present, while in the remaining 24 dispersion trials, agents differing in appearance (different hair colors and clothing) were presented. Furthermore, dispersion agents all had a neutral facial expression and did not direct their gaze at the participants. The dispersion trials were included in the experiment to hinder learning effects and to conceal the purpose of the study. The remaining 72 trials contained one of the four “target locations of interest” and were used for the later analysis. In 24 of those, no agent was presented, in 24 the presented agent had a neutral facial expression, and in 24 trials the presented agent had an angry facial expression (see Table 3). In trials without an agent, participants were instructed to directly look for the target and proceed as usual.

**Table 3:** Trial distribution.

	dispersion target	
agent present	yes	no
yes—neutral	24	24
yes—angry	-	24
no	12	24
	36	72

Distribution of the 108 trials, regarding agent presence and use of dispersion target. Only trials without a dispersion target were used for the later statistical analysis.

From the four targets of interest, two were presented on the left side of the room and two on the right side. The targets were always on the same side of the room as the agent. In regard to the agent’s position the target could either be close or far (see Fig 1). Close targets were positioned about 33 cm behind the agent’s position, they were 180.56 cm away from the start mark. The far targets were 66 cm away from the agent’s position and 206.14 cm away from the start mark.

### Evaluation of agents

After all trials had been executed, participants were asked to rate all used agents regarding valence (“How pleasant is this person?”), angriness (“How angry is this person?”) and realness (“How real is this person?”) with Likert scale items ranging from 1 (“not at all”) to 9 (“very much”). The agents appeared in a randomized order one after the other, once with a neutral and once with an angry facial expression. The prerecorded rating questions were asked via loudspeakers and participants rated by giving verbal response. Participants rated the agents via verbal response.

### Data reduction and statistical analyses

For the primary analysis the data for each trial was reduced to the time from target presentation to the point where the participant reached the target position and pressed the button. For a secondary explorative analysis, the data prior to the target presentation was extracted and analyzed as well. Several dependent variables were analyzed, from three different domains—distance, movement and pseudo-gaze direction (for reason of simplicity hereafter just called gaze direction). Regarding the secondary analysis, it is important to note, that its results are interpreted separately, as they were obtained during direct social interaction.

The distance domain includes two dependent variables. First, the kept minimum distance (**distance minimum**) to the center of the position where agents could appear. Second, the average distance (**distance average**) to the same position over all samples of the whole movement time.

In the movement domain, the **movement time** and the **movement speed** were analyzed. Movement time is the time participants needed for moving from the start position (starting with the target presentation) to standing on the target location. Movement speed is the average speed participants developed in that same time window. Finally, the gaze domain consists of three dependent variables. In this domain the vector for the direction participants were facing was calculated, using the head tracking data. Then a percentage score was calculated on how many times during a trial this vector hit the body of the agent (excluding head) or the head of the agent, resulting in the first two dependent variables: **gaze hits body** and **gaze hits head**. For the third variable, **gaze angle**, the angular distance from the direct gaze at the agents’ head was calculated as well and again averaged over the number of sample points [44]. For the secondary analysis, gaze hits body, gaze hits head, and the gaze angle were additionally calculated in the time window between start of trial and target presentation. The parameters described above were averaged for each participant and each condition and then analyzed with mixed repeated measures analyses of variance (ANOVAs). For the movement and distance domain, the ANOVAs consisted of the between-subjects factor **group** (HSA vs. control) and the within-subject factors **target position** (close vs. far) and **condition** (neutral agent vs. angry agent vs. none). This resulted in a 2 x 2 x 3 mixed repeated measures design. The within-subject factor condition was reduced to agent’s **expression** (neutral agent vs. angry agent) for analysis of the gaze domain. Also reducing the design to a 2 x 2 x 2 mixed repeated measures design. This was done, because without the agent, there is nothing to gaze at in the none condition. For parameters registered before target presentation, as analyzed in the secondary analysis, only the factors group and expression were added to the statistical analysis, resulting in a 2 x 2 mixed repeated measures design. T-tests were used to follow-up significant interactions. All statistical analyses used the two tailed 5% level of statistical significance, and all t-test p values were corrected using false discovery rate (FDR) correction [57]. When the assumption of sphericity was violated, results were corrected using Greenhouse-Geiser.

## Results

In this section, the results of the statistical analysis will be described. Figures of all described results can be found in the support information section.

### Distance domain

The distance average ANOVA resulted in a significant group x target interaction (*F*(1, 48) = 5.54, *p* = .023, $\eta_{p}^{2}=.10$). Post-hoc analysis showed that this was due to all participants keeping the same average distance with far targets (M_diff_ = 1.18 cm, *t*(56) = 1.08, *p* = 0.29), but HSA kept more distance than control with close targets (M_diff_ = 2.68 cm, *t*(56) = 2.44, *p* = .018). In addition, the ANOVA returned a significant target x condition interaction effect (*F*(1.67, 80.01) = 4.01, *p* = .028, $\eta_{p}^{2}=.08$). Here the subsequent analysis showed that no condition effect was present for close targets (angry—neutral: M_diff_ = 0.61 cm, *t*(160) = 1.43, *p* = .231; angry—none: M_diff_ = 0.68 cm, *t*(160) = 1.61, *p* = .231; neutral—none: M_diff_ = 0.07 cm, *t*(160) = 0.17, *p* = .862). In contrast, when targets were far, post hoc analysis revealed, that participants kept on average more distance when an agent was present (angry—none: M_diff_ = 0.87 cm, *t*(160) = 2.04, *p* = .064; neutral—none: M_diff_ = 1.25 cm, *t*(160) = 2.95, *p* = .011). However, the expression of the agent had no influence on the kept distance (angry—neutral: M_diff_ = 0.38 cm, *t*(160) = 0.91, *p* = .367). The distance minimum analysis revealed an interaction effect of target and condition (*F*(1.63, 78.03) = 14.08, *p* < .001, $\eta_{p}^{2}=.23$). Post hoc analysis showed that the minimal distance was smaller, when there was no agent for close targets (angry—none: M_diff_ < 16.81 cm, *t*(121) = 16.76, *p* < .001; neutral—none: M_diff_ = 16.55 cm, *t*(121) = 16.50, *p* < .001) and far targets (angry—none: M_diff_ < 13.49 cm, *t*(121) = 13.45, *p* < .001; neutral—none: M_diff_ = 13.59 cm, *t*(121) = 13.55, *p* < .001), but no difference for facial expressions, nor for close targets (angry—neutral: M_diff_ = 0.26 cm, *t*(121) = 0.80, *p* = .797), nor for far targets (angry—neutral: M_diff_ = 0.10 cm, *t*(121) = 0.10, *p* = .924).

### Movement domain

The ANOVA on movement time returned a significant target x condition interaction effect (*F*(2, 96) = 16.04, *p* < .001, $\eta_{p}^{2}=.25$). Post hoc analyses, revealed no significant effects between the three conditions for far targets (angry—neutral: M_diff_ = 37.88 ms, *t*(171) = 0.78, *p* = .436; angry—none: M_diff_ = 81.51 ms, *t*(171) = 1.68, *p* = .285; neutral—none: M_diff_ = 43.63 ms, *t*(171) = 0.90, *p* = .436). The analysis for close targets showed that participants were faster when there was no agent present (angry—none: M_diff_ = 259.37 ms, *t*(171) = 5.34, *p* < .001; neutral—none: M_diff_ = 356.73 ms, *t*(171) = 7.35, *p* < .001). In addition, participants needed less time to reach the close target position, when agents had an angry facial expression, compared to neutral agents (angry—neutral: M_diff_ = 97.36 ms, *t*(171) = 2.01, *p* = .046). The ANOVA also showed a marginal significant main effect of group (*F*(1, 48) = 3.55, *p* = .066, $\eta_{p}^{2}=.07$), with HSA needing less time to reach the target position (M_diff_ = 310.78 ms). The ANOVA on movement speed returned a significant three-way interaction effect of group x target x condition (*F*(2, 96) = 5.40, *p* < .006, $\eta_{p}^{2}=.10$). For post hoc analysis separate ANOVAs for close and far targets, with the factors group and condition, were conducted. Results for far targets showed a main effect of group (*F*(1, 48) = 4.25, *p* < .045, $\eta_{p}^{2}=.08$), with HSA developing higher average speed, than control participants (M_diff_ = 3.22 cm/s). The ANOVA on close targets returned a marginal significant interaction effect of group x condition (*F*(1.36, 65.28) = 3.16, *p* < .067, $\eta_{p}^{2}=.06$). Further post hoc analysis indicated that HSA were faster than controls, when an agent was present (angry: M_diff_ = 4.04 cm/s, *t*(63) = 2.57, *p* = .013; neutral: M_diff_ = 3.85 cm/s, *t*(63) = 2.45, *p* = .017). However, the control participants and HSA had the same speed without an agent (none: M_diff_ = 1.79 cm/s, *t*(63) = 1.14, *p* = .260).

### Gaze domain

Analysis of the gaze hits body variable returned a significant three-way interaction of group x expression x target (*F*(1, 48) = 8.18, *p* = .006, $\eta_{p}^{2}=.15$). Separate ANOVAs, with factors group and expression, were conducted for close and far target data. While no differences were found for far targets, the analysis of close target data returned a significant interaction effect of group x expression (*F*(1, 48) = 6.08, *p* = .017, $\eta_{p}^{2}=.11$). Further post hoc analysis showed that this interaction stems from control participants directing their gaze more at the body of the angry agent, when the target was close compared to far (M_diff_ = 0.82%, *t*(48) = 2.01, *p* = .051). HSA showed no differences regarding agent expression in their gazing behavior (M_diff_ = 0.61%, *t*(48) = 1.48, *p* = .145). The analysis of gaze hits head returned a main effect for target (*F*(1, 48) = 23.42, *p* < .001, $\eta_{p}^{2}=.33$), as participants directed their gaze at the agents’ head more often when targets were close (M_diff_ = 2.33%). In addition, this ANOVA showed a marginal significant main effect for expression (*F*(1, 48) = 3.42, *p* = .070, $\eta_{p}^{2}=.07$) suggesting that participants directed their gaze more towards the agents’ head when showing an angry expression (M_diff_ = 0.40%). The ANOVA on gaze angle returned a significant interaction effect for expression x target (*F*(1, 48) = 6.23, *p* = .016, $\eta_{p}^{2}=.11$). Post hoc analysis indicates opposing effects between facial expressions of close and far targets. The angular distance was bigger for neutral facial expressions than angry expressions, when participants had to get close (M_diff_ = 0.66°, *t*(88) = 2.19, *p* = .031). However, when the target position was far, angular distance was bigger, when agents had an angry facial expression, compared to a neutral one (M_diff_ = 0.55°, *t*(88) = 1.83, *p* = .071). In other words, angry facial expressions were approached, when participants had to get close to the agent, but were avoided when they could keep a bigger distance.

#### Gaze before target presentation

Analysis of gaze hits body before target presentation revealed an interaction of group x expression (*F*(1, 48) = 4.61, *p* = .039, $\eta_{p}^{2}=.09$). Control participants gazed more towards the body of the agent when he had an angry facial expression (M_diff_ = 1.35%, *t*(48) = 2.26, *p* = .029), while HSA participants did not (M_diff_ = 0.45%, *t*(48) = 0.75, *p* = .457). The ANOVA for gaze hits head returned a significant main effect for group (*F*(1, 48) = 4.49, *p* = .039, $\eta_{p}^{2}=.09$) and for expression (*F*(1, 48) = 6.98, *p* = .011, $\eta_{p}^{2}=.13$). Here HSA showed clear avoidance behavior by directing their gaze about 7.47% less often at the head of the agent compared to controls. In contrast, all participants focused their gaze more at the agent’s head when the facial expression of the agent was angry compared to neutral (M_diff_ = 1.29%). Finally, the analysis on gaze angle before target presentation did not return any significant effects.

### Evaluation of agents

Means and standard deviations of ratings for HSA and controls are presented in Table 4. Independent of group, agents with an angry compared to a neutral expression were perceived as less pleasant (*F*(1, 48) = 116.76, *p* < .001, $\eta_{p}^{2}=.71$). For the evaluated angriness, the ANOVA returned a significant group x agent expression interaction effect (*F*(1, 48) = 5.00, *p* < .030, $\eta_{p}^{2}=.09$). Post hoc analysis showed, that there is no group difference for the rating of agents with neutral expressions (M_diff_ = 0.2, *t*(94) = 0.60, *p* = .547). However, HSA gave higher angriness ratings for agents with angry expressions as controls (M_diff_ = 0.8, *t*(94) = 2.33, *p* = .022). Regarding the perceived realness of the agent the ANOVA showed a marginal significant main effect of expression (*F*(1, 48) = 3.21, *p* = .079, $\eta_{p}^{2}=.06$), indicating that agents with angry facial expressions were perceived as less real (M_diff_ = 0.2).

**Table 4:** Agent evaluation.

	HSA				control			
	neutral		angry		neutral		angry	
Question	*M*	*SD*	*M*	*SD*	*M*	*SD*	*M*	*SD*
How pleasant?	6.5	1.3	4.0	1.2	6.2	1.2	4.2	1.2
How angry?	1.6	0.6	6.2	1.6	1.8	0.6	5.4	1.5
How real?	6.1	1.4	5.9	1.5	6.3	1.7	6.2	1.6

Rating scores for agents with neutral and angry facial expressions. As rated by high socially anxious (HSA) and control participants.

## Discussion

The present study examined whether high socially anxious individuals differ in whole-body behavior when bypassing another person, here virtual humans with neutral and angry facial expressions, on the way to a specific target.

The first hypothesis predicted that virtual bystanders displaying angry facial expressions would be generally avoided. The conducted experiment showed evidence that this is true. When participants had to get close to the angry agents, they needed less time to complete trials, indicated by shorter trial times for close targets. Contrary to the hypothesized behavior, but in line with results from the study by McCall and colleagues [44], the participants gaze was directed generally more to angry compared to neutral faces. This reflects findings from non-VR studies, that emotional expressions attract and hold attention [58–61]. The observed differences in physical avoidance behavior between target conditions can be attributed to the targets being in two distinct zones related to different forms of personal space, as described by Hall [62]. The far target corresponded to the personal distance zone (46 to 76 cm) which is described as distance for interactions between friends or family members. The close target falls into the intimate distance zone, which is meant for close contact, such as embracing and touching. As both examined zones are reserved for friends and family members, one would expect, that an angry stranger would elicit larger interpersonal distance in both zones. However, it seems that due to the task requesting participants to overcome those urges several times an extreme closeness like the intimate zone must be reached to elicit clear differences. Alternatively, it may be speculated that behaviors towards virtual persons differ in this respect from real persons. Further studies are needed, which systematically look at interpersonal distance zones and task compliance with analyses of progression of speed and distance while participants are approaching the different zones. The present study focused on facial expressions as these are very relevant social signs affecting behavior which can be modulated in virtual reality with high ecological validity. However, other important signs of social interaction, e.g. body postures and movements, should be examined in future studies as these variables have a demonstrated relevance for social cognition (e.g. Teneggi *et al*. [63]).

Results also confirmed the second hypothesis, as HSA displayed generally enhanced avoidance behavior towards agents. Overall, they spend less time in the social situation because of a higher movement speed, as HSA were especially faster than controls, when an agent was present. Furthermore, and in line with results by Wieser *et. al*. [29], HSA kept more interpersonal distance, when they had to get close to the agent (close targets). No group differences regarding the attention towards agents, indicated by gazing behavior, were detected after target presentation. This is most likely due to the fact, that the task directed attention away from the agent anyhow. When examining the time before target presentation, which was initially no objective of the study, it was revealed that HSA gazed less at the head of the agent confirming the expected avoidance of gaze contact related to HSA. As mentioned before, it must be kept in mind that before target presentation the participants shortly engaged in an explicit social interaction as they were asked to name the agent’s hair color. The difference in gaze data may therefore be a consequence of this explicitly required interaction.

The study failed to support the last hypothesis that avoidance behavior of socially anxious participants is specifically exaggerated towards an agent with a negative facial expression. Only gazing behavior towards the body of the angry agent showed group differences. Here control participants exhibited more attention towards the angry agent’s body than towards the neutral agent’s body, while HSA displayed no statistically significant differences in this behavior. One explanation of this could be that, in addition to looking more at the head of angry agents, control participants also look at the body for further clues on the opponent’s emotional state and HSA do not. This could be explained, by the fact that people suffering SAD tend to allocate attention largely away from the social situation towards themselves, as stated by the two leading cognitive models on SAD from Clark and Wells [24] and Rapee and Heimberg [27]. Overall, the mentioned restrictions of the task could be one reason for the low levels of variation between groups. Furthermore, participants executed the task with ease and were very accurate in positioning themselves on the target position. In a further study, the task should be more difficult, to increase the level of variation and thereby the possible influence of social factors. One way to increase difficulty would be to increase the time between target presentation and start of movement. Another reason for the lack of exaggerated avoidance behavior of HSA towards angry agents could be that, although the agents were rated as angry, facial expressions were not aversive enough. In future research it would be advisable to further add an objective measure for the valence of the virtual person, for example, measuring skin conductance, which has been shown to be sensitive for approach-avoidance in VR experiments [64, 65].

To our knowledge this is the first study which used immersive VR with a CAVE setup to investigate social whole-body approach-avoidance behavior in HSA. As stated before, the study successfully replicated prior findings and the newly developed VR task proved to be a valid tool for research on approach-avoidance behavior. In line with other studies [29, 44, 64] it showed that VR allows research with high ecologically and internal validity. Furthermore, to our knowledge this is the first study on social approach-avoidance behavior in VR in which the agent was irrelevant for the task and where action or attention towards the agent was completely voluntary. The CAVE system, on the one hand, allows an ecological valid simulation and quite natural movement with perception of the own body. On the other hand, the restricted dimension of the CAVE (4 x 3 m) only allows restricted movement distances, in this study max. 2 m, which weakens ecological validity. Future studies might use recent developments of improved and wireless HMD systems, which however also allow tracking in an area of 4 x 4 m only. In addition, HMDs have a crucial downside hampering ecological validity as participants cannot see their own body. In the future, virtual bodies might be simulated, however, future studies need to clarify whether a simulated artificial body has ecological validity. Next to the different ways on how to use the VR social approach-avoidance task in research projects, it could for example also be used as behavioral avoidance test (BAT), to get an objective measure of social anxiety. This could be useful for assessing SAD on a behavioral level for long term studies on SAD treatment and as an objective measure for therapeutic success.

Some remaining limitations and methodological difficulties of the described study, need be further addressed in future research. First, our participants were mainly female. This likely was due to the screening for social anxiety, as females have a higher prevalence for anxiety disorders [66, 67]. However, the prevalence for social phobia is still discussed and seems to show no gender differences [67–69]. Another reason might be that the screened population consisted mainly of psychology students, which are more often female. In addition to the gender bias for the participants, the agents used were all male. Future research should investigate, whether gender of the participant and the agent has an effect on the social approach and avoidance behavior. Another possible limitation of the study is the usage of head orientation as factor for where participants directed attention. Here the measurement of eye movement would be the more precious method. However, head orientation alone has an accuracy of 88.7% to detect the focus of attention [70]. Furthermore, since it seems relevant for SAD whether someone directly looks at you or not, it would be interesting for future studies to investigate the impact of direct gaze on approach-avoidance behavior.

## Supporting information

S1 Fig Mean and standard deviation of percentage gaze directed at agent body per trial, for group (HSA and control), regarding different facial expressions (angry and neutral). (# *p* < .1, * *p* < .05, ** *p* < .01, *** *p* < .001).
(TIF)

S2 Fig Mean and SDs of average distance to agent position in cm, for condition (angry agent, neutral agent and none), regarding target (close and far). (# *p* < .1, * *p* < .05, ** *p* < .01, *** *p* < .001).
(TIF)

S3 Fig Mean and SDs of minimum distance to agent position in cm, for condition (angry agent, neutral agent and none), regarding target (close and far). (# *p* < .1, * *p* < .05, ** *p* < .01, *** *p* < .001).
(TIF)

S4 Fig Mean and SDs in seconds, of time participants needed for moving from the start position to standing on the target location for condition (angry agent, neutral agent, and none), regarding target position (close and far). (# *p* < .1, * *p* < .05, ** *p* < .01, *** *p* < .001).
(TIF)

S5 Fig Mean and SDs in seconds, of time participants needed for moving from the start position to standing on the target location for group (HAS and control). (# *p* < .1, * *p* < .05, ** *p* < .01, *** *p* < .001).
(TIF)

S6 Fig Mean and SDs of average speed in cm/s, for group (HSA and control), regarding different conditions (angry agent, neutral agent, and none) for trials with close targets. (# *p* < .1, * *p* < .05, ** *p* < .01, *** *p* < .001).
(TIF)

S7 Fig Mean and SDs of average speed in cm/s, for different conditions (angry agent, neutral agent, and none), regarding group (HSA and control) for trials with far targets. (# *p* < .1, * *p* < .05, ** *p* < .01, *** *p* < .001).
(TIF)

S8 Fig Mean and SDs in percentage head directed at agent body per trial, for group (HSA and control), regarding different facial expressions (angry and neutral) for trials with close and far targets separately. (# *p* < .1, * *p* < .05, ** *p* < .01, *** *p* < .001).
(TIF)

S9 Fig Mean and SDs of percentage gaze directed at agent head per trial, for target position (close and far). (# *p* < .1, * *p* < .05, ** *p* < .01, *** *p* < .001).
(TIF)

S10 Fig Mean and SDs of percentage gaze directed at agent head per trial, for expression (angry and neutral). (# *p* < .1, * *p* < .05, ** *p* < .01, *** *p* < .001).
(TIF)

S11 Fig Mean and SDs of average angular distance from the direct gaze at the agents’ head per trial, for expression (angry and neutral), regarding target position (close and far). (# *p* < .1, * *p* < .05, ** *p* < .01, *** *p* < .001).
(TIF)

S12 Fig Mean and SDs of percentage gaze directed at agent body before target presentation, for group (HSA and control), regarding different facial expressions (angry and neutral). (# *p* < .1, * *p* < .05, ** *p* < .01, *** *p* < .001).
(TIF)

S13 Fig Mean and SDs of percentage gaze directed at agent head before target presentation, for group (HSA and control). (# *p* < .1, * *p* < .05, ** *p* < .01, *** *p* < .001).
(TIF)

S14 Fig Mean and SDs of percentage gaze directed at agent head before target presentation, for expression (angry and neutral). (# *p* < .1, * *p* < .05, ** *p* < .01, *** *p* < .001).
(TIF)

S15 Fig Mean and SDs of valence ratings of the agents (“How pleasant is this person”) with Likert scale items ranging from 1 (“not at all”) to 9 (“very much”). (# *p* < .1, * *p* < .05, ** *p* < .01, *** *p* < .001).
(TIF)

S16 Fig Mean and SDs of angriness ratings of the agents (“How angry is this person?”) with Likert scale items ranging from 1 (“not at all”) to 9 (“very much”). (# *p* < .1, * *p* < .05, ** *p* < .01, *** *p* < .001).
(TIF)

S17 Fig Mean and SDs of realness ratings of the agents (“How real is this person?”) with Likert scale items ranging from 1 (“not at all”) to 9 (“very much”). (# *p* < .1, * *p* < .05, ** *p* < .01, *** *p* < .001).
(TIF)

## References

[1] Lang PJ, Bradley MM. Emotion and the motivational brain. Biological psychology. 2010;84(3):437–450. 10.1016/j.biopsycho.2009.10.007 19879918 PMC3612949

[2] Phaf RH, Mohr SE, Rotteveel M, Wicherts JM. Approach, avoidance, and affect: a meta-analysis of approach-avoidance tendencies in manual reaction time tasks. Frontiers in psychology. 2014;5:378 10.3389/fpsyg.2014.00378 24847292 PMC4021119

[3] Chen M, Bargh JA. Consequences of Automatic Evaluation: Immediate Behavioral Predispositions to Approach or Avoid the Stimulus. Personality and Social Psychology Bulletin. 1999;25(2):215–224. 10.1177/0146167299025002007

[4] Eder AB, Rothermund K. When do motor behaviors (mis)match affective stimuli? An evaluative coding view of approach and avoidance reactions. Journal of experimental psychology General. 2008;137(2):262–281. 10.1037/0096-3445.137.2.262 18473659

[5] de Houwer J, Crombez G, Baeyens F, Hermans D. On the generality of the affective Simon effect. Cognition & Emotion. 2001;15(2):189–206. 10.1080/02699930125883

[6] Markman AB, Brendl CM. Constraining theories of embodied cognition. Psychological science. 2005;16(1):6–10. 10.1111/j.0956-7976.2005.00772.x 15660844

[7] Rinck M, Becker ES. Approach and avoidance in fear of spiders. Journal of behavior therapy and experimental psychiatry. 2007;38(2):105–120. 10.1016/j.jbtep.2006.10.001 17126289

[8] Saraiva AC, Schuur F, Bestmann S. Emotional valence and contextual affordances flexibly shape approach-avoidance movements. Frontiers in psychology. 2013;4:933 10.3389/fpsyg.2013.00933 24379794 PMC3861787

[9] Seidel EM, Habel U, Kirschner M, Gur RC, Derntl B. The impact of facial emotional expressions on behavioral tendencies in women and men. Journal of experimental psychology Human perception and performance. 2010;36(2):500–507. 10.1037/a0018169 20364933 PMC2852199

[10] Marsh AA, Ambady N, Kleck RE. The effects of fear and anger facial expressions on approach- and avoidance-related behaviors. Emotion (Washington, DC). 2005;5(1):119–124. 10.1037/1528-3542.5.1.119 15755225

[11] Roelofs K, Elzinga BM, Rotteveel M. The effects of stress-induced cortisol responses on approach-avoidance behavior. Psychoneuroendocrinology. 2005;30(7):665–677. 10.1016/j.psyneuen.2005.02.008 15854783

[12] Stins JF, Roelofs K, Villan J, Kooijman K, Hagenaars MA, Beek PJ. Walk to me when I smile, step back when I’m angry: emotional faces modulate whole-body approach-avoidance behaviors. Experimental brain research. 2011;212(4):603–611. 10.1007/s00221-011-2767-z 21698468 PMC3133774

[13] Laham SM, Kashima Y, Dix J, Wheeler M. A meta-analysis of the facilitation of arm flexion and extension movements as a function of stimulus valence. Cognition & Emotion. 2015;29(6):1069–1090. 10.1080/02699931.2014.968096 25345558

[14] Heuer K, Rinck M, Becker ES. Avoidance of emotional facial expressions in social anxiety: The Approach-Avoidance Task. Behaviour research and therapy. 2007;45(12):2990–3001. 10.1016/j.brat.2007.08.010 17889827

[15] Roelofs K, van Peer J, Berretty E, Jong Pd, Spinhoven P, Elzinga BM. Hypothalamus-pituitary-adrenal axis hyperresponsiveness is associated with increased social avoidance behavior in social phobia. Biological psychiatry. 2009;65(4):336–343. 10.1016/j.biopsych.2008.08.022 18947821

[16] Roelofs K, Putman P, Schouten S, Lange WG, Volman I, Rinck M. Gaze direction differentially affects avoidance tendencies to happy and angry faces in socially anxious individuals. Behaviour research and therapy. 2010;48(4):290–294. 10.1016/j.brat.2009.11.008 19962692

[17] Fehm L, Pelissolo A, Furmark T, Wittchen HU. Size and burden of social phobia in Europe. European neuropsychopharmacology: the journal of the European College of Neuropsychopharmacology. 2005;15(4):453–462. 10.1016/j.euroneuro.2005.04.002 15921898

[18] Furmark T. Social phobia: Overview of community surveys. Acta Psychiatrica Scandinavica. 2002;105(2):84–93. 10.1034/j.1600-0447.2002.1r103.x 11939957

[19] Kessler RC, Berglund P, Demler O, Jin R, Merikangas KR, Walters EE. Lifetime prevalence and age-of-onset distributions of DSM-IV disorders in the National Comorbidity Survey Replication. Archives of general psychiatry. 2005;62(6):593–602. 10.1001/archpsyc.62.6.593 15939837

[20] Ruscio AM, Brown TA, Chiu WT, Sareen J, Stein MB, Kessler RC. Social fears and social phobia in the USA: results from the National Comorbidity Survey Replication. Psychological medicine. 2008;38(1):15–28. 10.1017/S0033291707001699 17976249 PMC2262178

[21] American Psychiatric Association. Diagnostic and Statistical Manual of Mental Disorders: DSM-IV-TR. 4th ed Washington, DC: American Psychiatric Publishing, Inc.; 2000.

[22] American Psychiatric Association. Diagnostic and Statistical Manual of Mental Disorders: DSM-V. 5th ed Washington, DC: American Psychiatric Publishing, Inc.; 2013.

[23] Beck AT, Emery G, Greenberg RL. Anxiety disorders and phobias: A cognitive perspective. Basic Books; 1985.

[24] Clark DM, Wells A. A cognitive model of social phobia In: Social phobia: Diagnosis, assessment, and treatment. New York, NY, US: Guilford Press; 1995 p. 69–93.

[25] Eysenck MW, Derakshan N, Santos R, Calvo MG. Anxiety and cognitive performance: Attentional control theory. Emotion (Washington, DC). 2007;7(2):336–353. 10.1037/1528-3542.7.2.336 17516812

[26] Mathews A, Mackintosh B. A Cognitive Model of Selective Processing in Anxiety. Cognitive Therapy & Research. 1998;22(6):539–560. 10.1023/A:1018738019346

[27] Rapee RM, Heimberg RG. A cognitive-behavioral model of anxiety in social phobia. Behaviour Research and Therapy. 1997;35(8):741–756. 10.1016/s0005-7967(97)00022-3 9256517

[28] Beidel DC, Turner SM, Dancu CV. Physiological, cognitive and behavioral aspects of social anxiety. Behaviour Research and Therapy. 1985;23(2):109–117. 10.1016/0005-7967(85)90019-1 4004691

[29] Wieser MJ, Pauli P, Grosseibl M, Molzow I, Mühlberger A. Virtual social interactions in social anxiety–the impact of sex, gaze, and interpersonal distance. Cyberpsychology, behavior and social networking. 2010;13(5):547–554. 10.1089/cyber.2009.0432 20950179

[30] Koch S, Holland RW, Hengstler M, van Knippenberg A. Body locomotion as regulatory process: stepping backward enhances cognitive control. Psychological science. 2009;20(5):549–550. 10.1111/j.1467-9280.2009.02342.x 19476588

[31] Adler LL, Iverson MA. Interpersonal distance as a function of task difficulty, praise, status orientation, and sex of partner. Perceptual and Motor Skills. 1974;39(2):683–692. 10.2466/pms.1974.39.2.683

[32] Aiello JR. A further look at equilibrium theory: Visual interaction as a function of interpersonal distance. Environmental Psychology and Nonverbal Behavior. 1977;1(2):122–140. 10.1007/BF01145461

[33] Uzzell D, Horne N. The influence of biological sex, sexuality and gender role on interpersonal distance. The British journal of social psychology / the British Psychological Society. 2006;45(Pt 3):579–597. 10.1348/014466605X58384 16984722

[34] Hayduk LA. Personal space: Where we now stand. Psychological Bulletin. 1983;94(2):293–335. 10.1037/0033-2909.94.2.293

[35] Bombari D, Schmid Mast M, Canadas E, Bachmann M. Studying social interactions through immersive virtual environment technology: Virtues, pitfalls, and future challenges. Frontiers in psychology. 2015;6:869 10.3389/fpsyg.2015.00869 26157414 PMC4478377

[36] Kuhlen AK, Brennan SE. Language in dialogue: When confederates might be hazardous to your data. Psychonomic bulletin & review. 2013;20(1):54–72. 10.3758/s13423-012-0341-8 23188738

[37] Blascovich J, Loomis J, Beall AC, Swinth KR, Hoyt CL, Bailenson JN. Immersive Virtual Environment Technology as a Methodological Tool for Social Psychology. Psychological Inquiry. 2002;13(2):103–124. 10.1207/S15327965PLI1302_01

[38] McCall C, Blascovich J. How, When, and Why to Use Digital Experimental Virtual Environments to Study Social Behavior. Social and Personality Psychology Compass. 2009;3(5):744–758. 10.1111/j.1751-9004.2009.00195.x

[39] Parsons TD, Gaggioli A, Riva G. Virtual Reality for Research in Social Neuroscience. Brain sciences. 2017;7(4). 10.3390/brainsci7040042 28420150 PMC5406699

[40] Garau M, Slater M, Pertaub DP, Razzaque S. The Responses of People to Virtual Humans in an Immersive Virtual Environment. Presence: Teleoperators and Virtual Environments. 2005;14(1):104–116. 10.1162/1054746053890242

[41] Hoyt CL, Blascovich J, Swinth KR. Social Inhibition in Immersive Virtual Environments. Presence: Teleoperators and Virtual Environments. 2003;12(2):183–195. 10.1162/105474603321640932

[42] James LK, Lin CY, Steed A, Swapp D, Slater M. Social anxiety in virtual environments: results of a pilot study. Cyberpsychology & behavior: the impact of the Internet, multimedia and virtual reality on behavior and society. 2003;6(3):237–243. 10.1089/109493103322011515 12855078

[43] Kane HS, McCall C, Collins NL, Blascovich J. Mere presence is not enough: Responsive support in a virtual world. Journal of Experimental Social Psychology. 2012;48(1):37–44. 10.1016/j.jesp.2011.07.001

[44] McCall C, Hildebrandt LK, Hartmann R, Baczkowski BM, Singer T. Introducing the Wunderkammer as a tool for emotion research: Unconstrained gaze and movement patterns in three emotionally evocative virtual worlds. Computers in Human Behavior. 2016;59:93–107. 10.1016/j.chb.2016.01.028

[45] Parsons TD, Rizzo AA. Affective outcomes of virtual reality exposure therapy for anxiety and specific phobias: a meta-analysis. Journal of behavior therapy and experimental psychiatry. 2008;39(3):250–261. 10.1016/j.jbtep.2007.07.007 17720136

[46] Rinck M, Rörtgen T, Lange WG, Dotsch R, Wigboldus DHJ, Becker ES. Social anxiety predicts avoidance behaviour in virtual encounters. Cognition & Emotion. 2010;24(7):1269–1276. 10.1080/02699930903309268

[47] Ahrens LM, Muhlberger A, Pauli P, Wieser MJ. Impaired visuocortical discrimination learning of socially conditioned stimuli in social anxiety. Social cognitive and affective neuroscience. 2015;10(7):929–937. 10.1093/scan/nsu140 25338634 PMC4483562

[48] Reutter M, Hewig J, Wieser MJ, Osinsky R. The N2pc component reliably captures attentional bias in social anxiety. Psychophysiology. 2017;54(4):519–527. 10.1111/psyp.12809 28111770

[49] Wieser MJ, Moscovitch DA. The Effect of Affective Context on Visuocortical Processing of Neutral Faces in Social Anxiety. Frontiers in psychology. 2015;6:1824 10.3389/fpsyg.2015.01824 26648889 PMC4663271

[50] Laux L, Glanzmann P, Schaffner P, Spielberger CD. Das State-Trait-Angstinventar (STAI): Theoretische Grundlagen und Handanweisung. Weinheim: Beltz Test GmbH; 1981.

[51] Bradley MM, Lang PJ. Measuring emotion: The self-assessment manikin and the semantic differential. Journal of behavior therapy and experimental psychiatry. 1994;25(1):49–59. 10.1016/0005-7916(94)90063-9 7962581

[52] Schubert T, Friedmann F, Regenbrecht H. Embodied Presence in Virtual Environments In: Paton R, Neilson I, editors. Visual Representations and Interpretations. London: Springer London; 1999 p. 269–278.

[53] Kennedy RS, Lane NE, Berbaum KS, Lilienthal MG. Simulator Sickness Questionnaire: An Enhanced Method for Quantifying Simulator Sickness. The International Journal of Aviation Psychology. 1993;3(3):203–220. 10.1207/s15327108ijap0303_3

[54] Fydrich T. SPAI—Soziale Phobie und Angst Inventar In: Brähler E, Schumacher J, Strauß B, editors. Diagnostische Verfahren in der Psychotherapie. Göttingen: Hogrefe; 2002 p. 335–338.

[55] Ekman P, Friesen WV. Manual for the Facial Action Coding System. Palo Alto, Calif.: Consulting Psychologists Press; 1978.

[56] Ekman P, Friesen WV, Hager JC. Facial action coding system. Salt Lake City, UT: A Human Face; 2002.

[57] Benjamini Y, Hochberg Y. Controlling the False Discovery Rate: A Practical and Powerful Approach to Multiple Testing. Journal of the Royal Statistical Society Series B (Methodological). 1995;57(1):289–300. 10.1111/j.2517-6161.1995.tb02031.x

[58] Batty M, Taylor MJ. Early processing of the six basic facial emotional expressions. Cognitive Brain Research. 2003;17(3):613–620. 10.1016/s0926-6410(03)00174-5 14561449

[59] Green MJ, Phillips ML. Social threat perception and the evolution of paranoia. Neuroscience and biobehavioral reviews. 2004;28(3):333–342. 10.1016/j.neubiorev.2004.03.006 15225975

[60] Lundqvist D, Juth P, Öhman A. Using facial emotional stimuli in visual search experiments: The arousal factor explains contradictory results. Cognition & Emotion. 2014;28(6):1012–1029. 10.1080/02699931.2013.867479 24341823

[61] Palermo R, Rhodes G. Are you always on my mind? A review of how face perception and attention interact. Neuropsychologia. 2007;45(1):75–92. 10.1016/j.neuropsychologia.2006.04.025 16797607

[62] Hall ET. The Hidden Dimension. Leonardo. 1966;6.

[63] Teneggi C, Canzoneri E, Di Pellegrino G, Serino A. Social modulation of peripersonal space boundaries. Current biology. 2013;23(5):406–411. 10.1016/j.cub.2013.01.043 23394831

[64] Dotsch R, Wigboldus DHJ. Virtual prejudice. Journal of Experimental Social Psychology. 2008;44(4):1194–1198. 10.1016/j.jesp.2008.03.003

[65] Wilcox LM, Allison RS, Elfassy S, Grelik C. Personal space in virtual reality. ACM Transactions on Applied Perception. 2006;3(4):412–428. 10.1145/1190036.1190041

[66] Cohen P, Cohen J, Kasen S, Velez CN, Hartmark C, Johnson J, et al An Epidemiological Study of Disorders in Late Childhood and Adolescence? I. Age- and Gender-Specific Prevalence. Journal of Child Psychology and Psychiatry. 1993;34(6):851–867. 10.1111/j.1469-7610.1993.tb01094.x 8408371

[67] McLean CP, Asnaani A, Litz BT, Hofmann SG. Gender differences in anxiety disorders: Prevalence, course of illness, comorbidity and burden of illness. Journal of psychiatric research. 2011;45(8):1027–1035. 10.1016/j.jpsychires.2011.03.006 21439576 PMC3135672

[68] Furmark T, Tillfors M, Everz PO, Marteinsdottir I, Gefvert O, Fredrikson M. Social phobia in the general population: Prevalence and sociodemographic profile. Social Psychiatry and Psychiatric Epidemiology. 1999;34(8):416–424. 10.1007/s001270050163 10501711

[69] Turk CL, Heimberg RG, Orsillo SM, Holt CS, Gitow A, Street LL, et al An Investigation of Gender Differences in Social Phobia. Journal of Anxiety Disorders. 1998;12(3):209–223. 10.1016/s0887-6185(98)00010-3 9653680

[70] Stiefelhagen R, Zhu J. Head orientation and gaze direction in meetings In: Terveen L, Wixon D, editors. CHI’02 extended abstracts on Human factors in computer systems—CHI’02. New York, New York, USA: ACM Press; 2002 p. 858.

