# Supplementary figures and images for: Social anxiety changes the way we move—A social approach-avoidance task in a virtual reality CAVE system
Source: PLoS One. 2019 Dec 23;14(12):e0226805. doi: 10.1371/journal.pone.0226805 (PMC6927627; doi:10.1371/journal.pone.0226805)

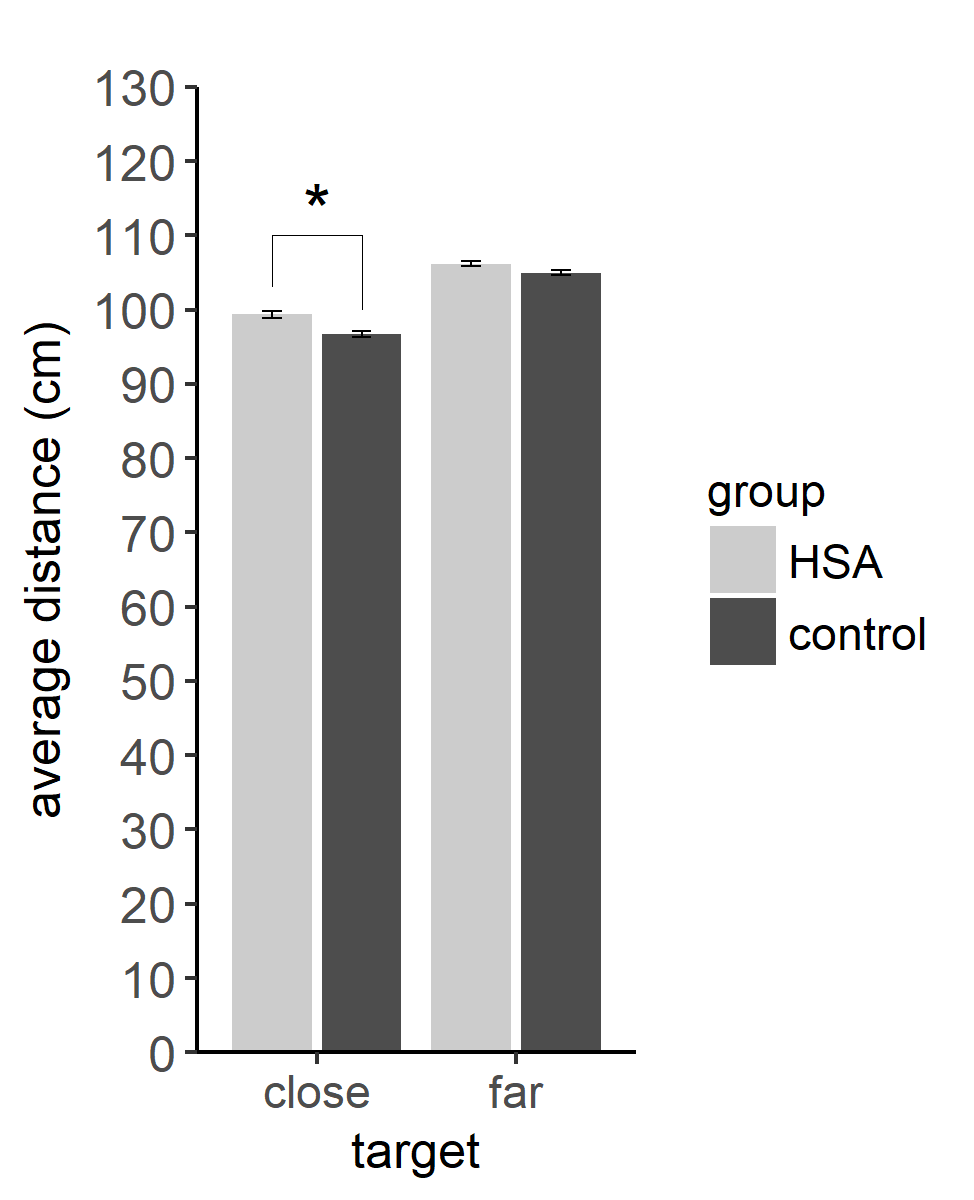

Supplement: S1 Fig — (# p < .1, * p < .05, ** p < .01, *** p < .001). (TIF) [file pone.0226805.s001.tif]

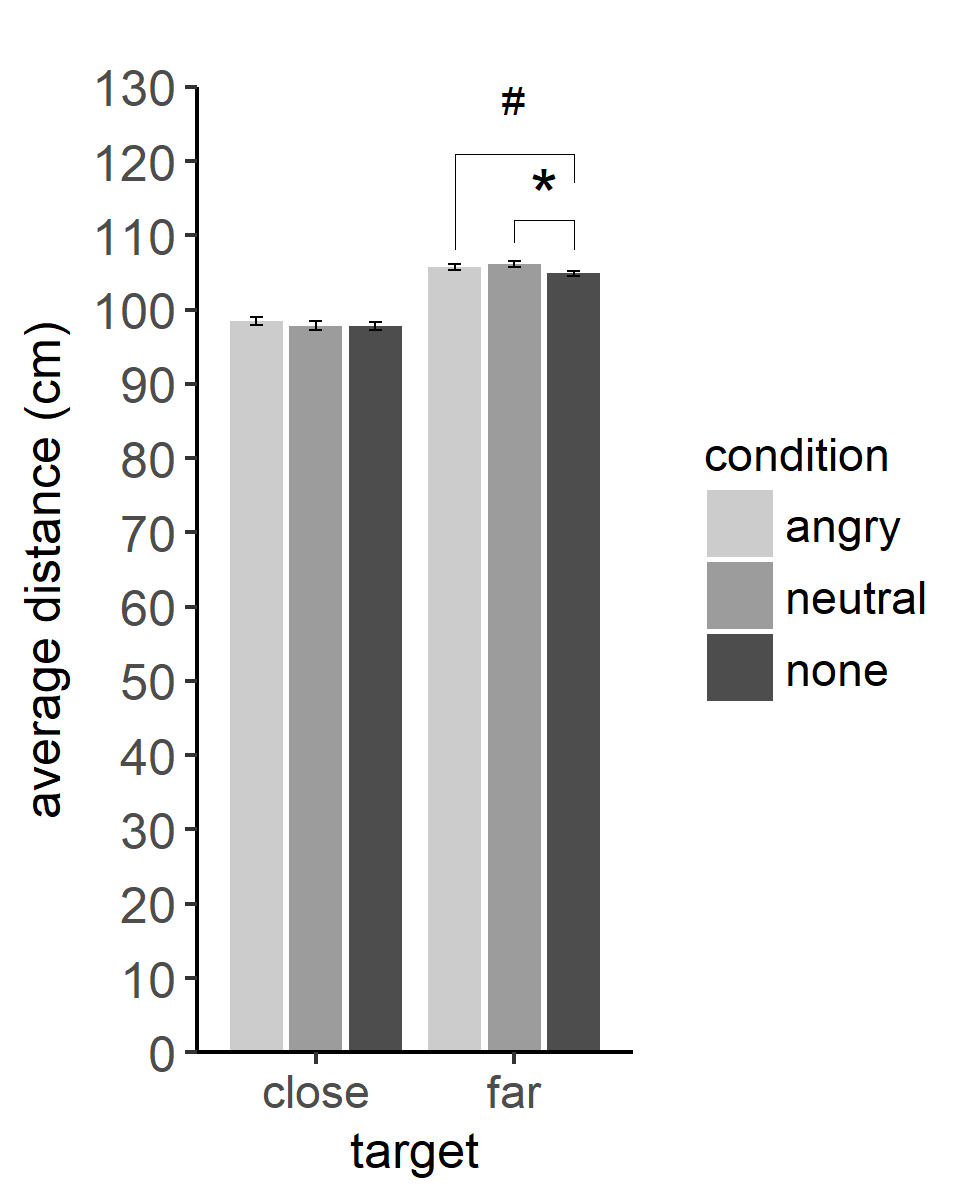

Supplement: S2 Fig — (# p < .1, * p < .05, ** p < .01, *** p < .001). (TIF) [file pone.0226805.s002.tif]

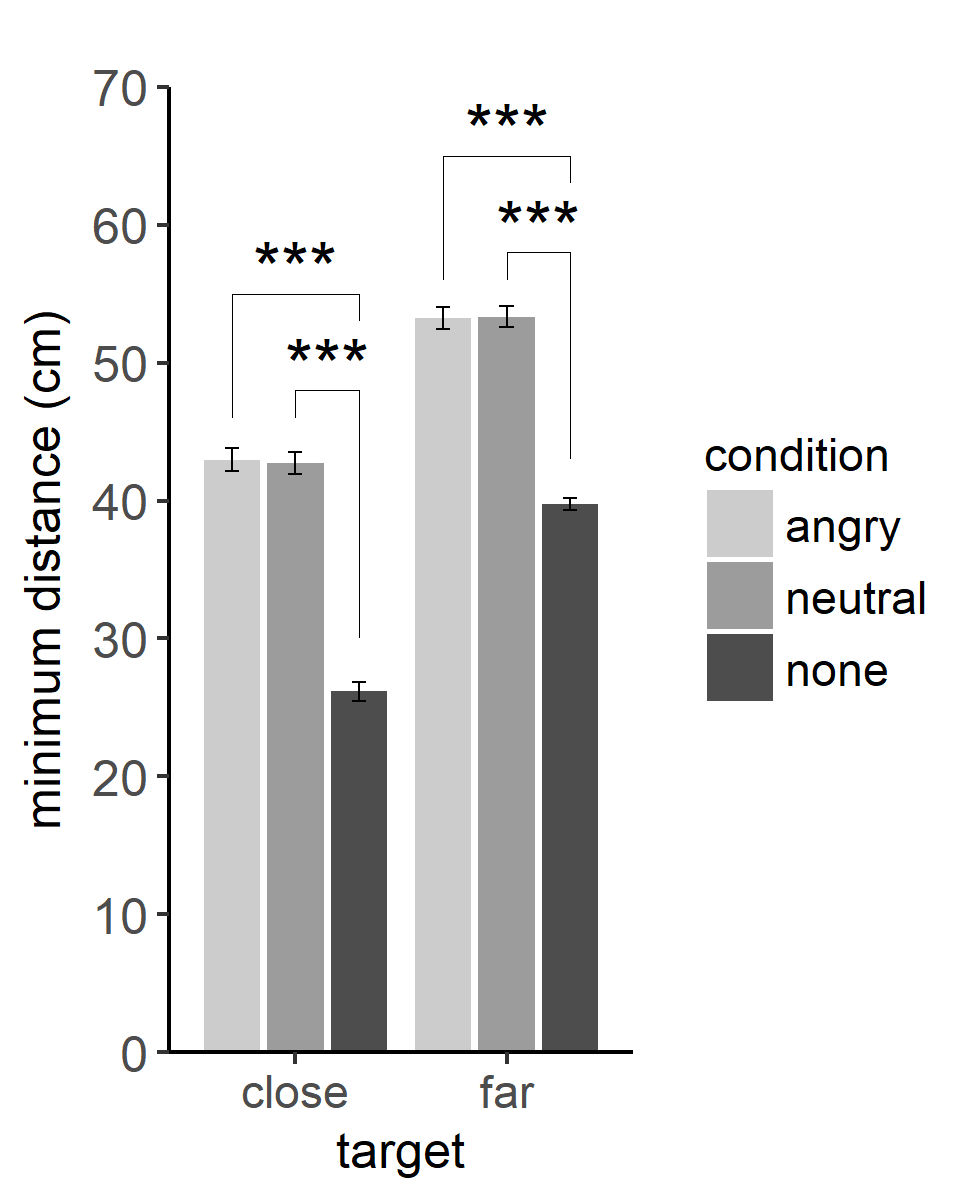

Supplement: S3 Fig — (# p < .1, * p < .05, ** p < .01, *** p < .001). (TIF) [file pone.0226805.s003.tif]

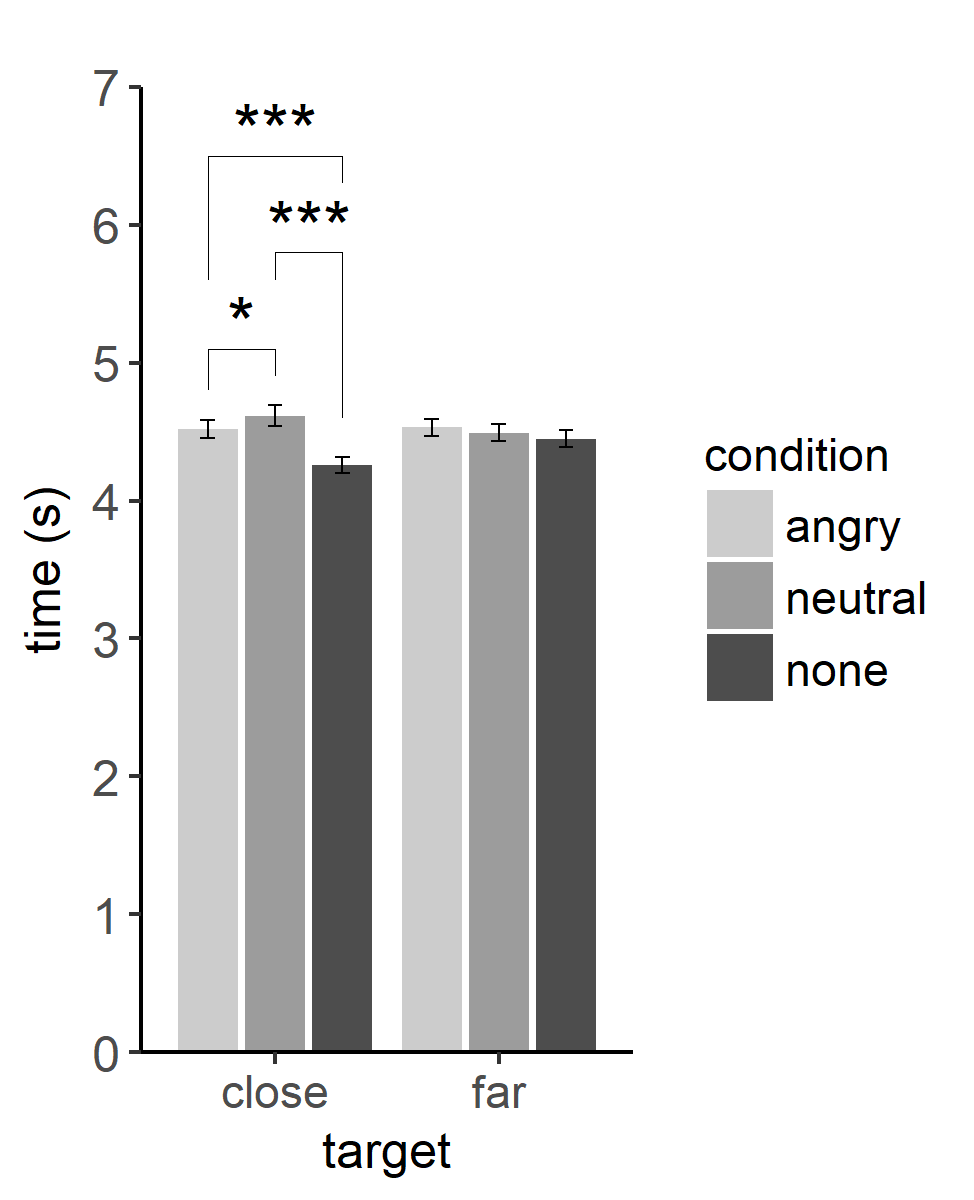

Supplement: S4 Fig — (# p < .1, * p < .05, ** p < .01, *** p < .001). (TIF) [file pone.0226805.s004.tif]

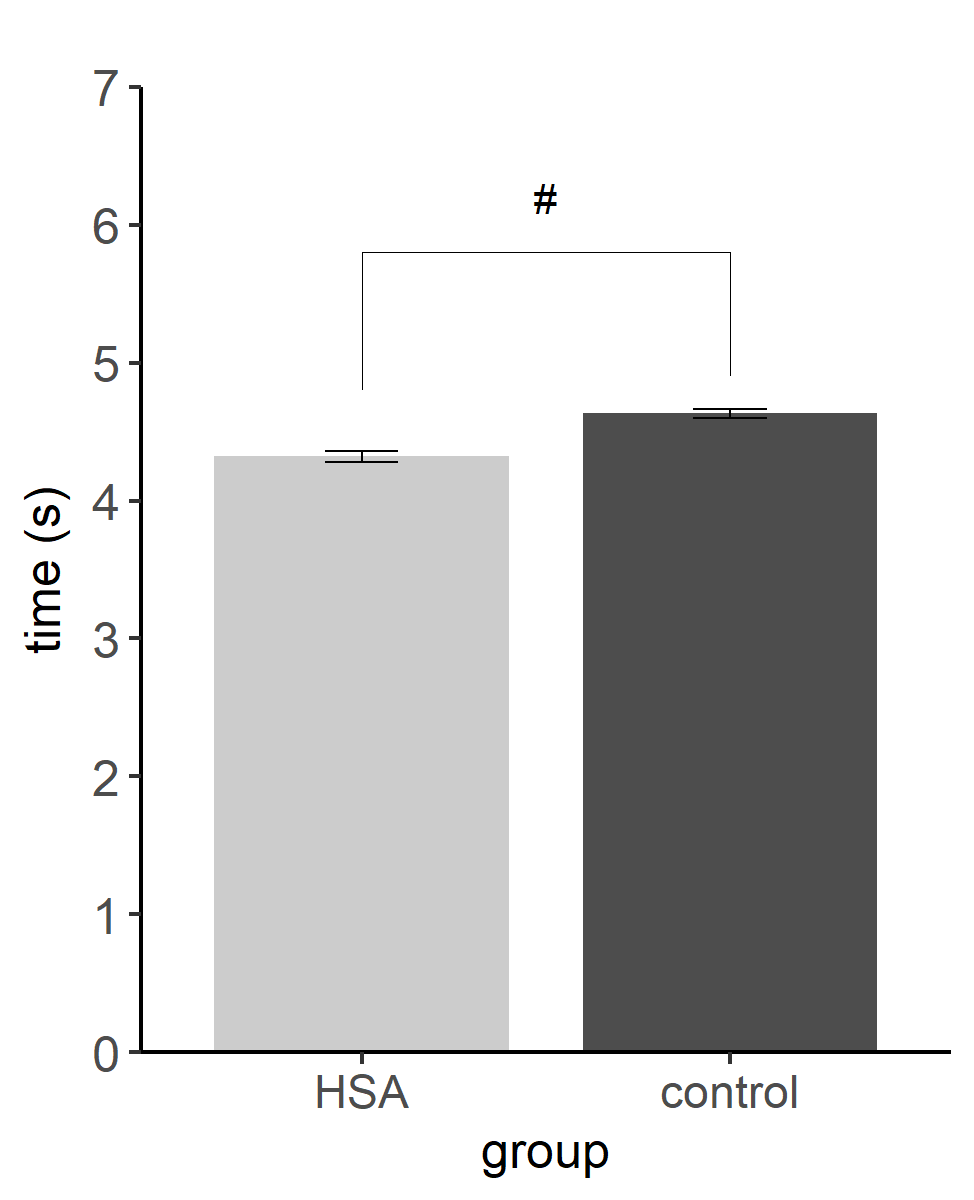

Supplement: S5 Fig — (# p < .1, * p < .05, ** p < .01, *** p < .001). (TIF) [file pone.0226805.s005.tif]

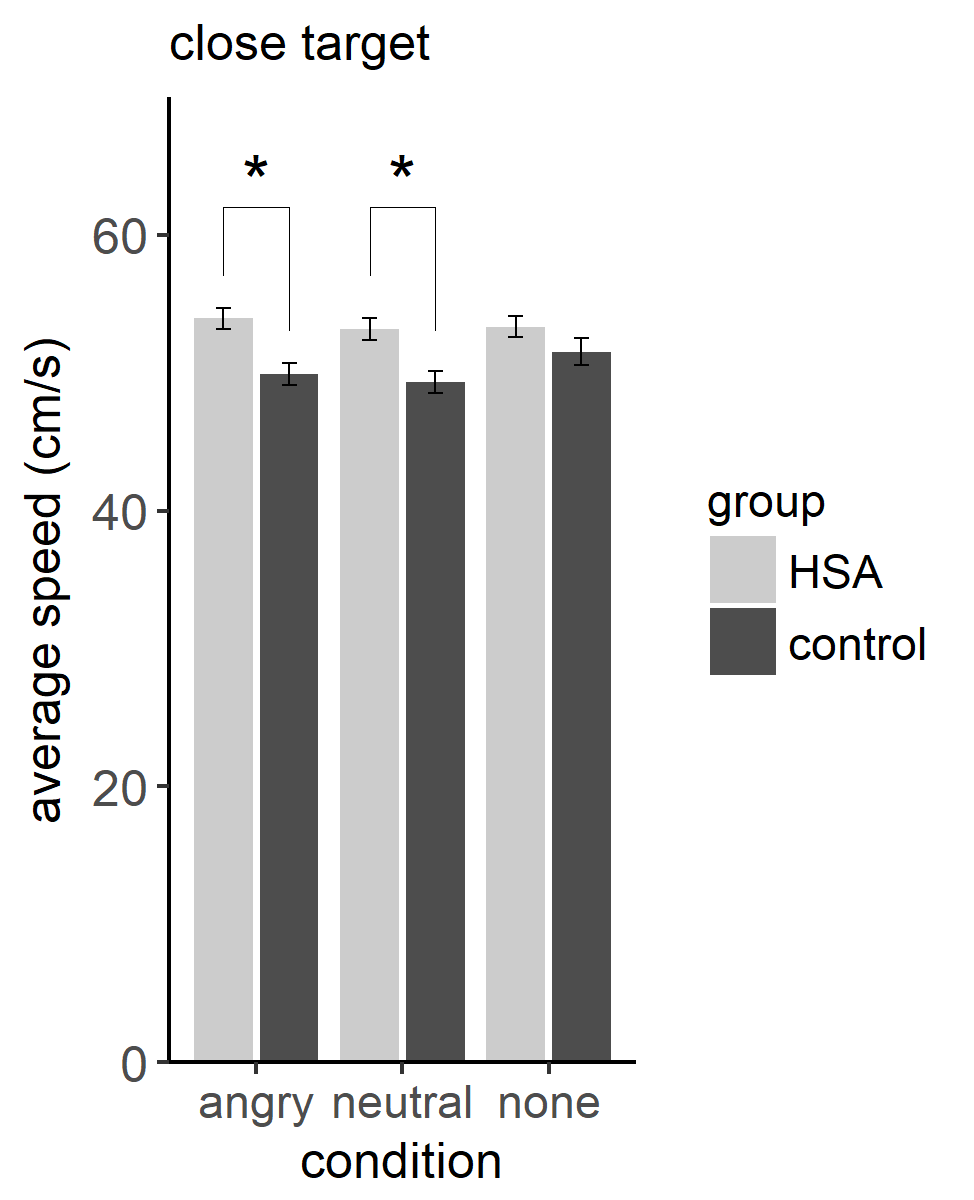

Supplement: S6 Fig — (# p < .1, * p < .05, ** p < .01, *** p < .001). (TIF) [file pone.0226805.s006.tif]

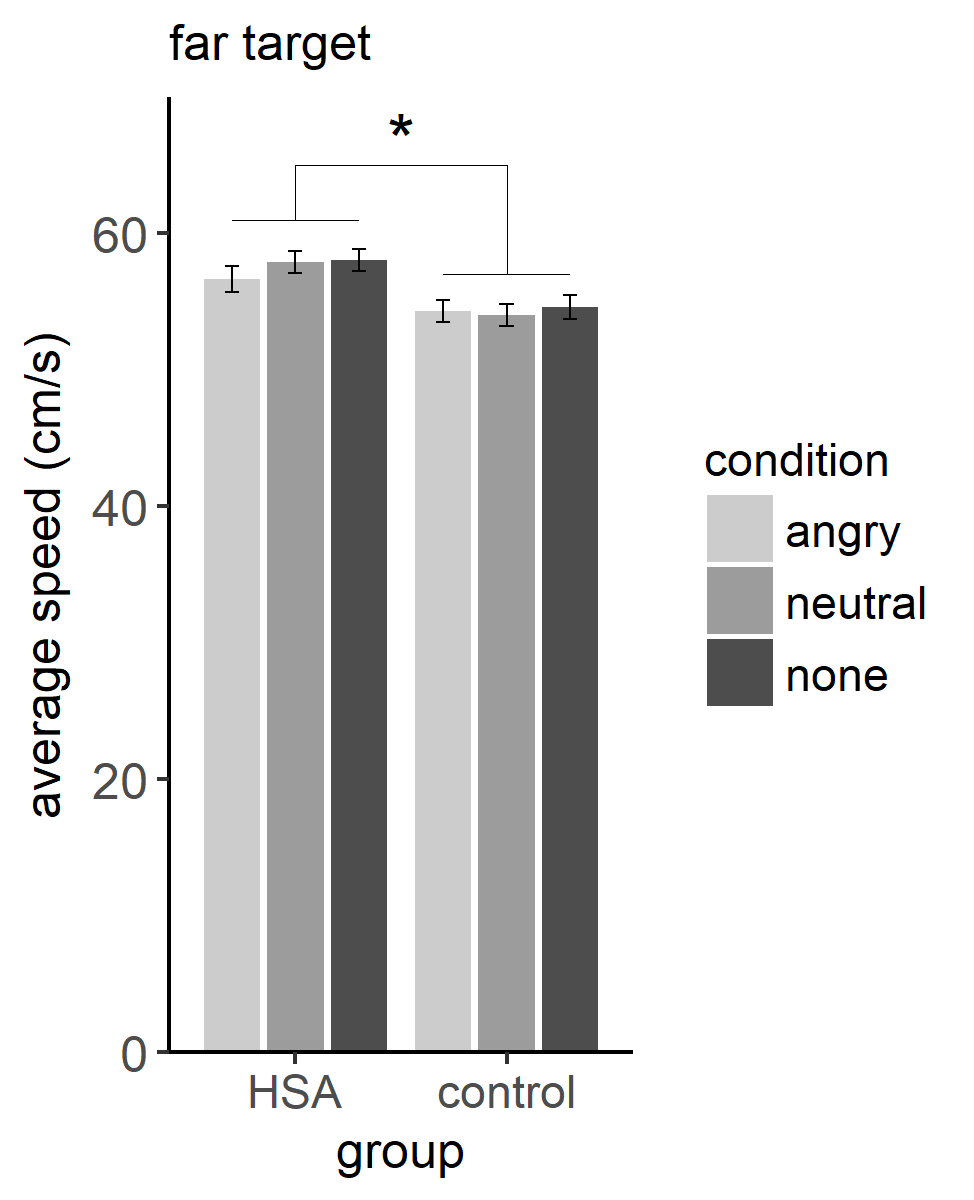

Supplement: S7 Fig — (# p < .1, * p < .05, ** p < .01, *** p < .001). (TIF) [file pone.0226805.s007.tif]

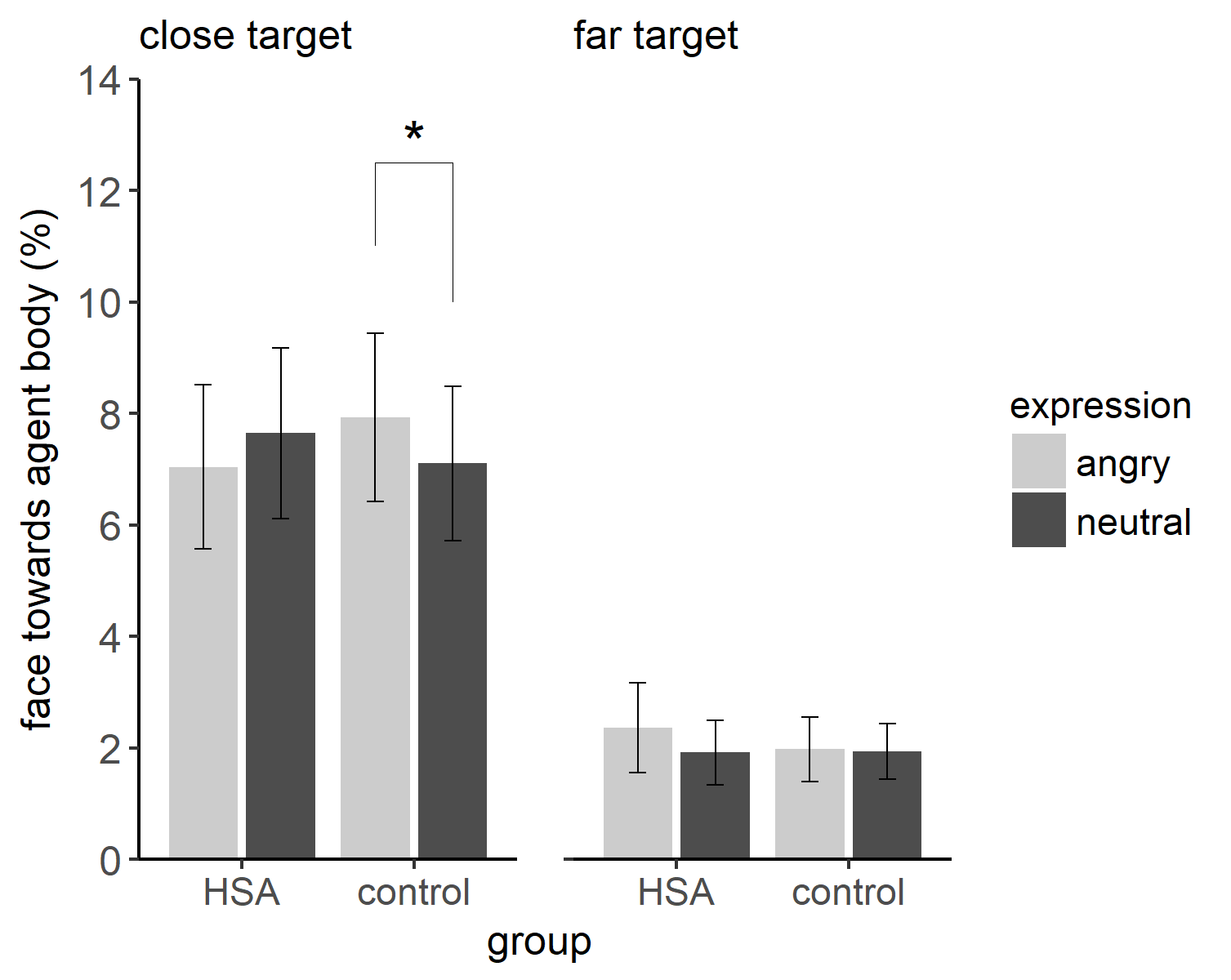

Supplement: S8 Fig — (# p < .1, * p < .05, ** p < .01, *** p < .001). (TIF) [file pone.0226805.s008.tif]

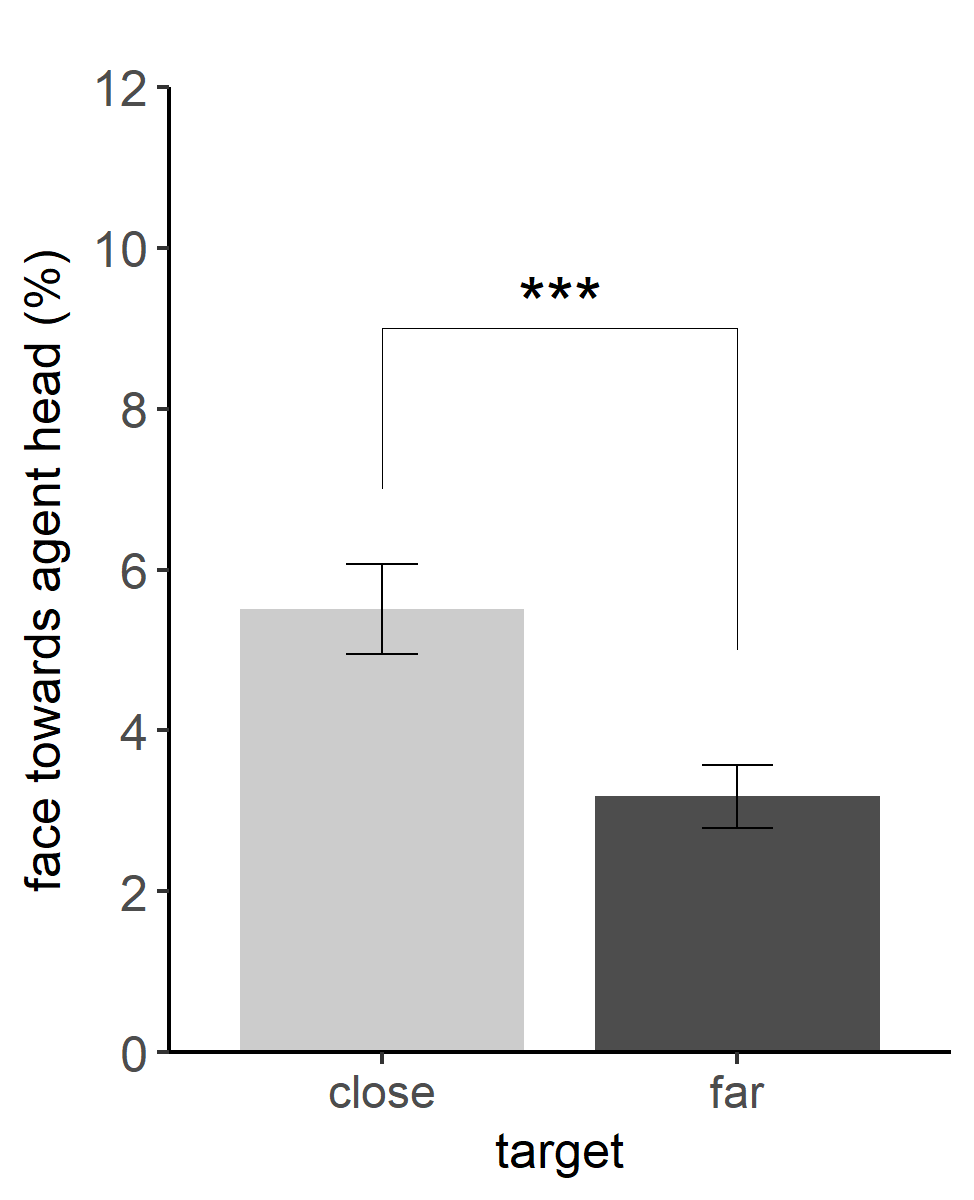

Supplement: S9 Fig — (# p < .1, * p < .05, ** p < .01, *** p < .001). (TIF) [file pone.0226805.s009.tif]

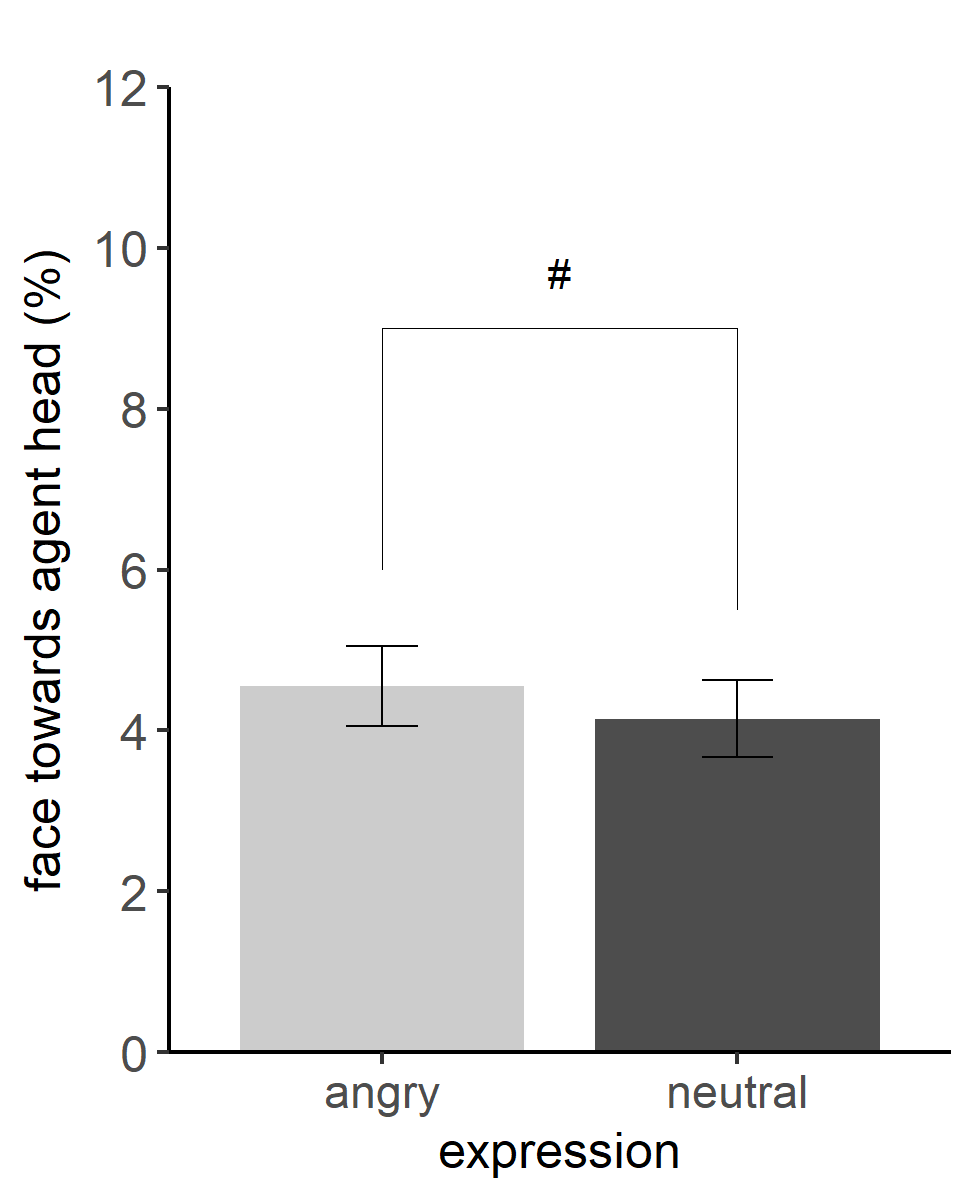

Supplement: S10 Fig — (# p < .1, * p < .05, ** p < .01, *** p < .001). (TIF) [file pone.0226805.s010.tif]

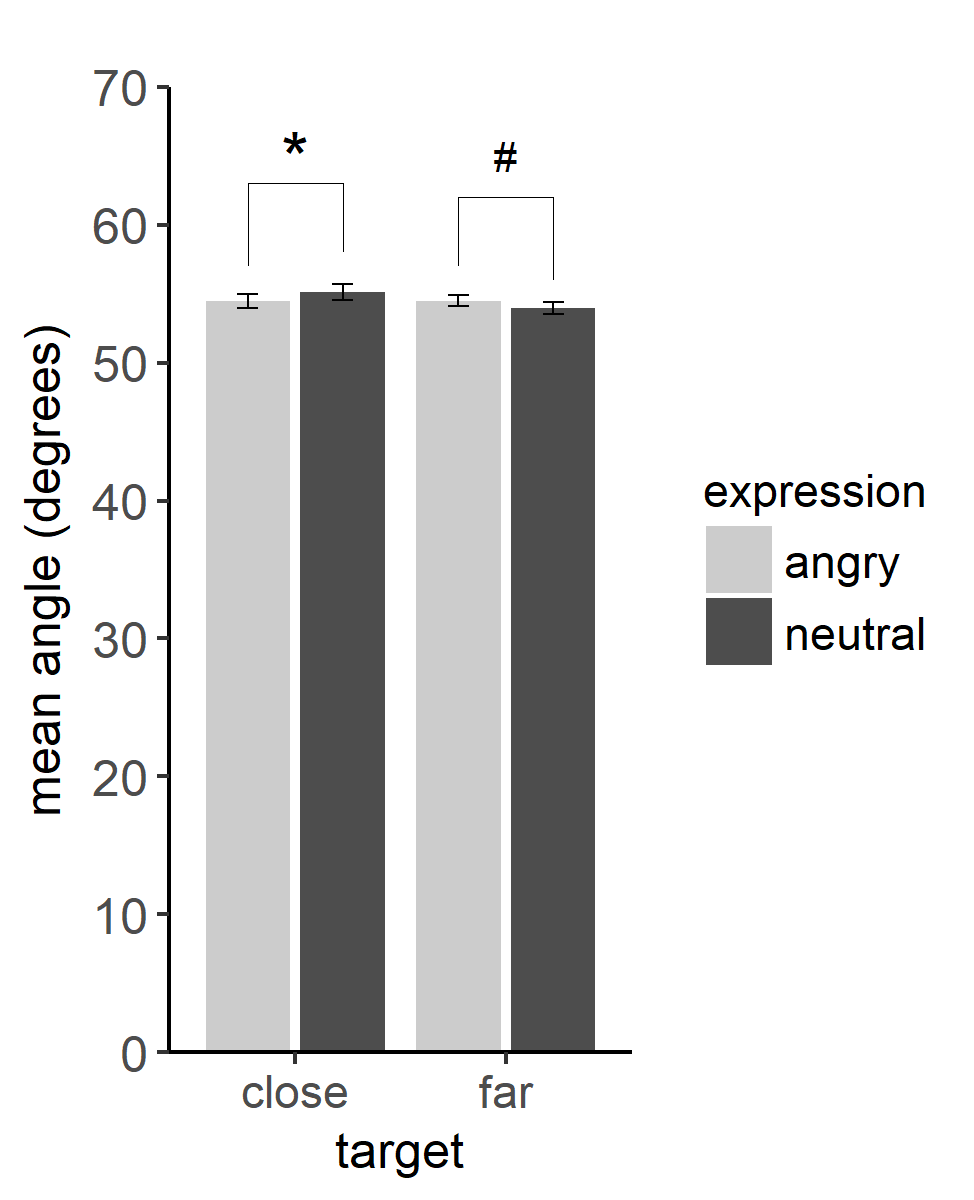

Supplement: S11 Fig — (# p < .1, * p < .05, ** p < .01, *** p < .001). (TIF) [file pone.0226805.s011.tif]

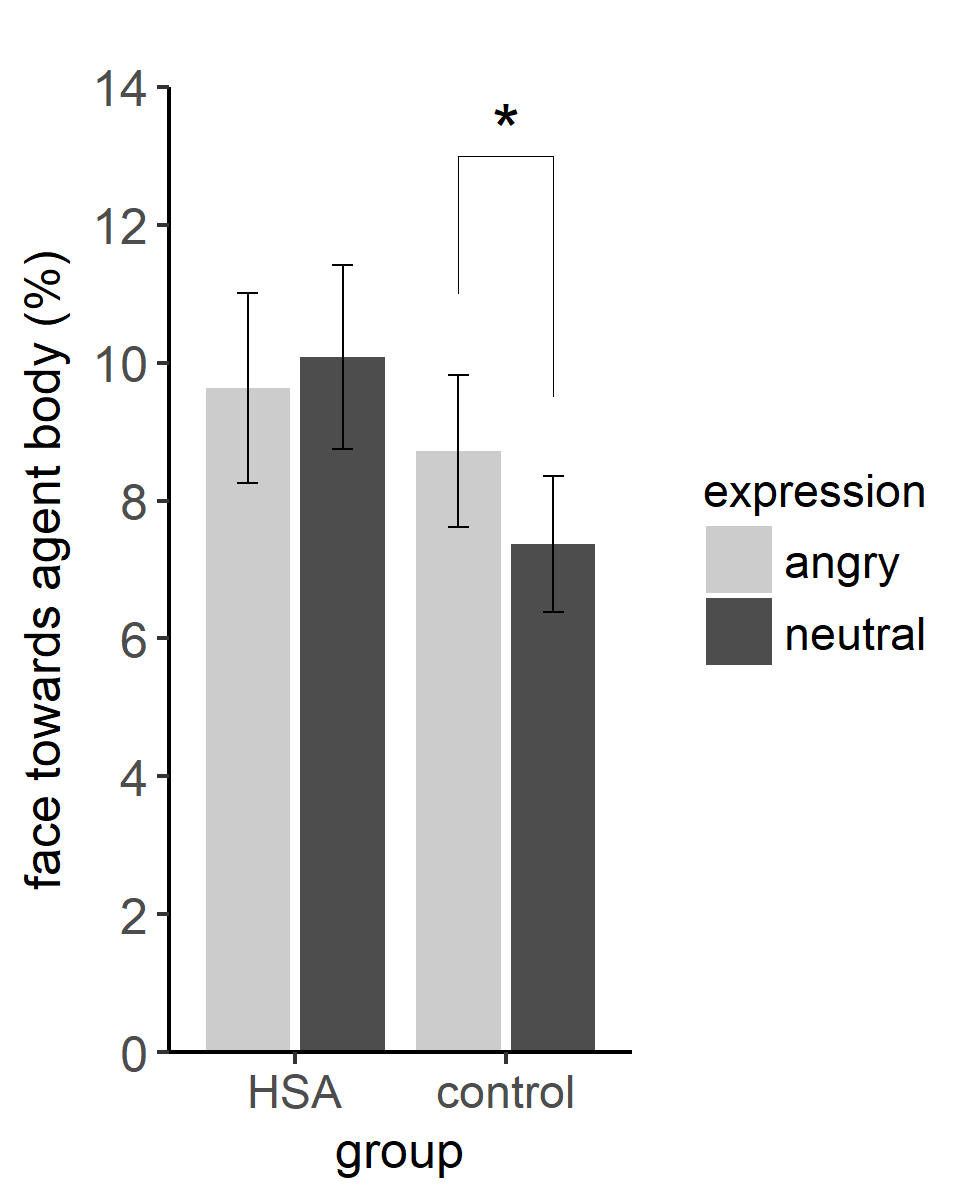

Supplement: S12 Fig — (# p < .1, * p < .05, ** p < .01, *** p < .001). (TIF) [file pone.0226805.s012.tif]

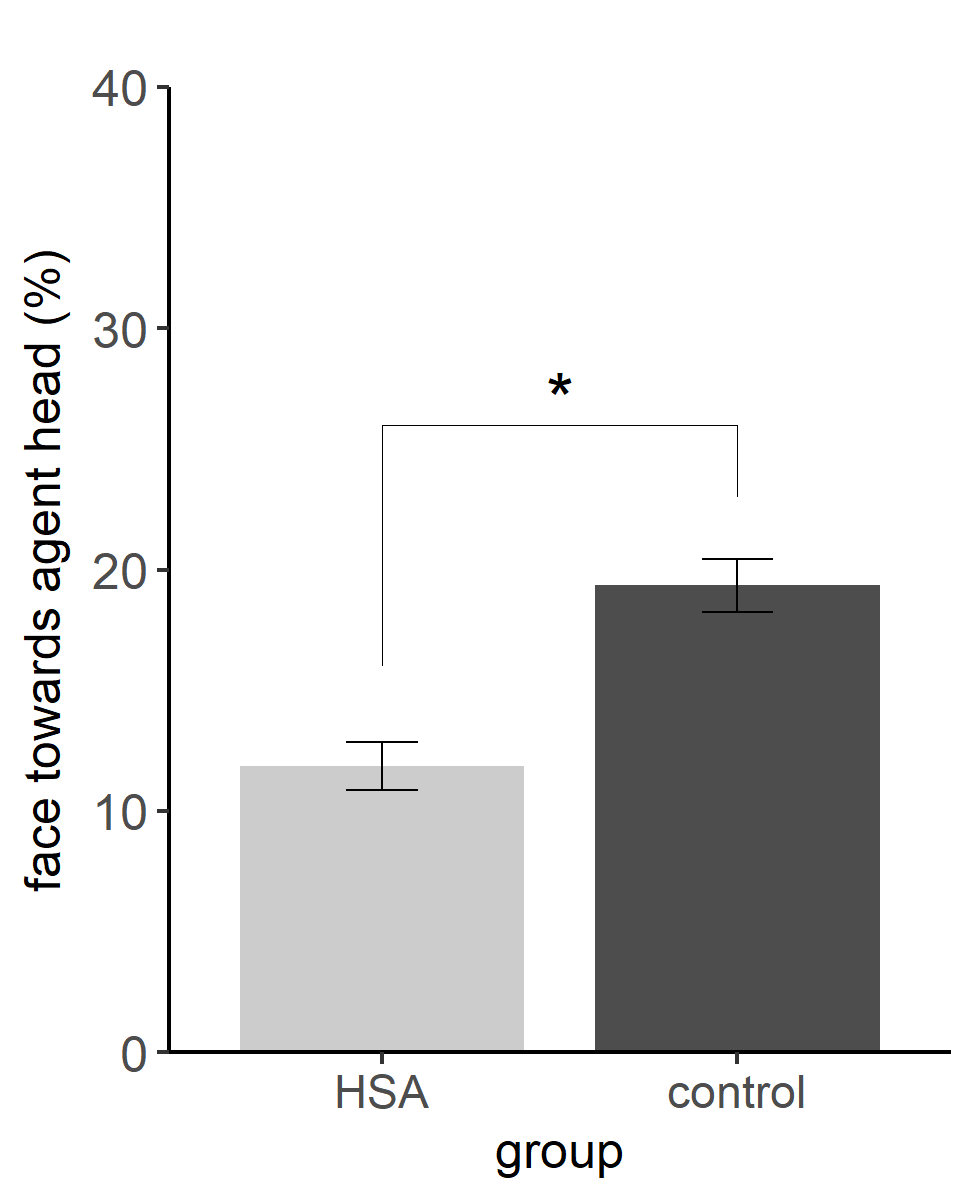

Supplement: S13 Fig — (# p < .1, * p < .05, ** p < .01, *** p < .001). (TIF) [file pone.0226805.s013.tif]

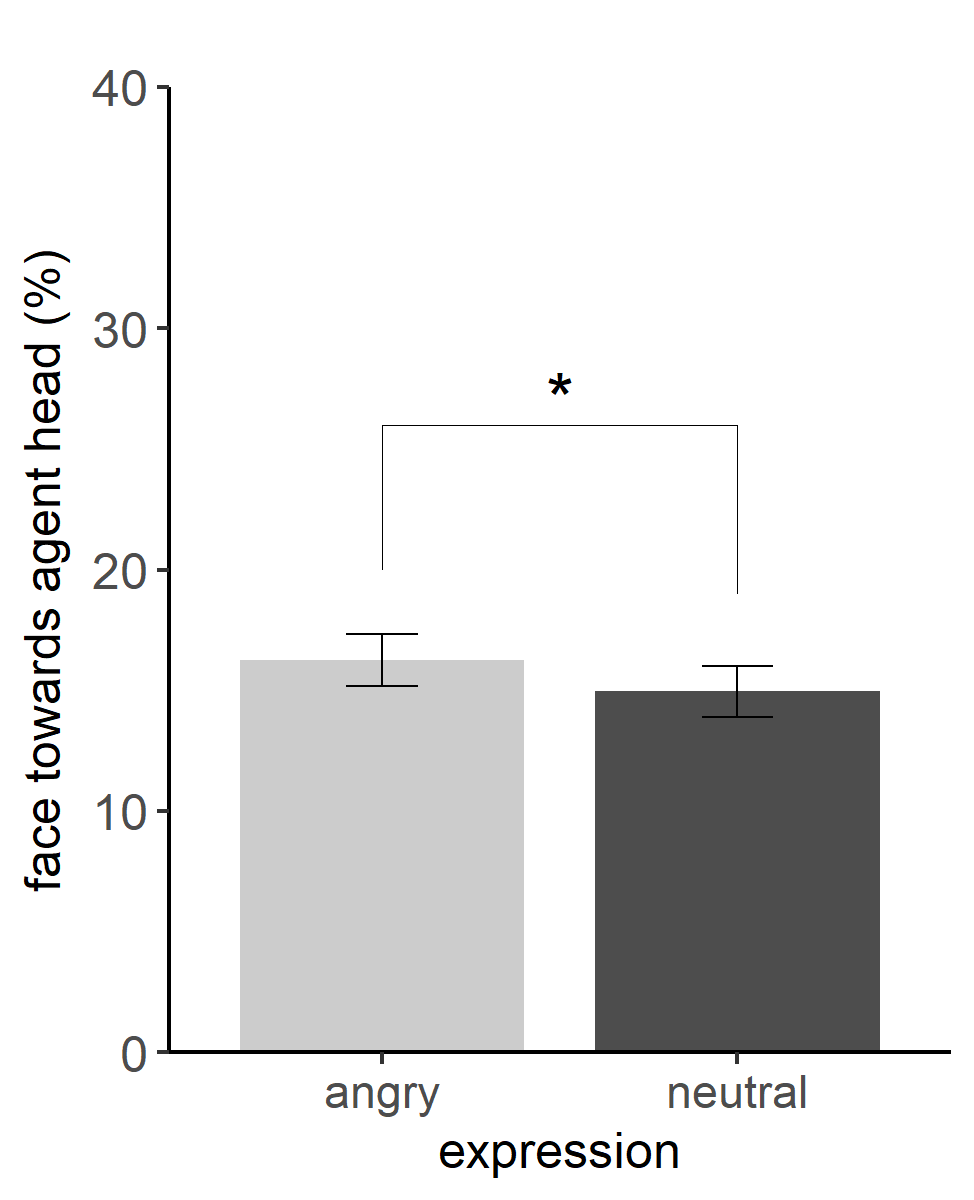

Supplement: S14 Fig — (# p < .1, * p < .05, ** p < .01, *** p < .001). (TIF) [file pone.0226805.s014.tif]

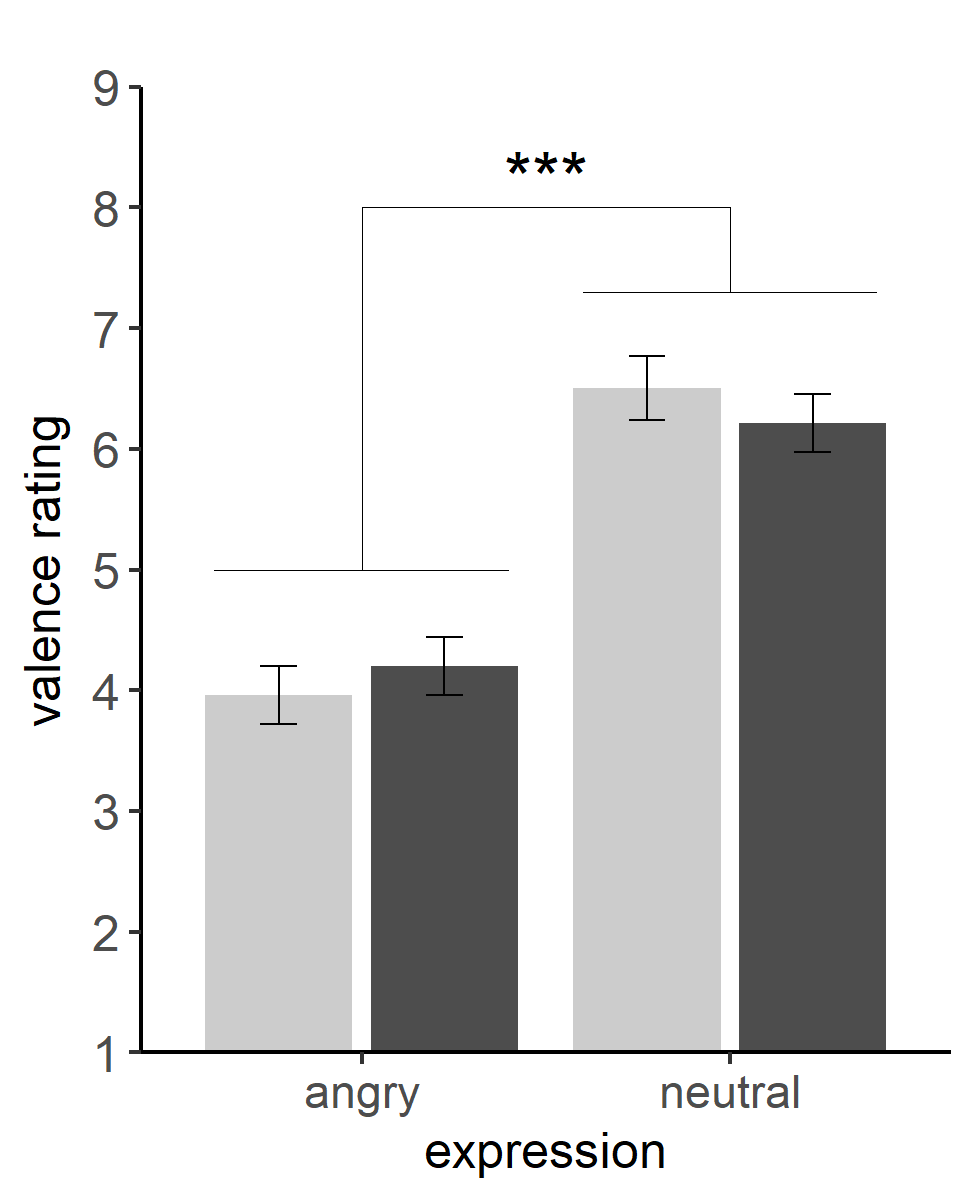

Supplement: S15 Fig — (# p < .1, * p < .05, ** p < .01, *** p < .001). (TIF) [file pone.0226805.s015.tif]

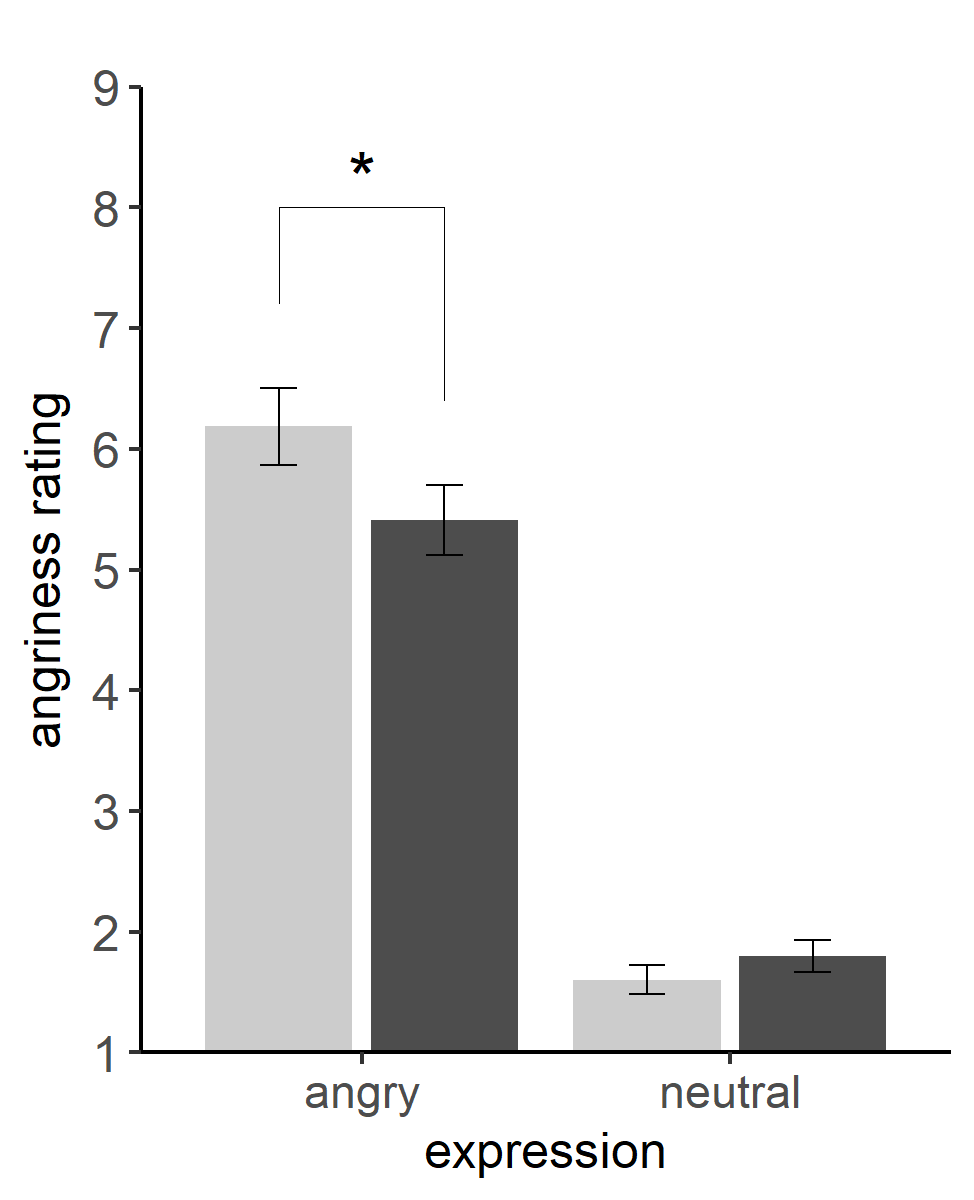

Supplement: S16 Fig — (# p < .1, * p < .05, ** p < .01, *** p < .001). (TIF) [file pone.0226805.s016.tif]

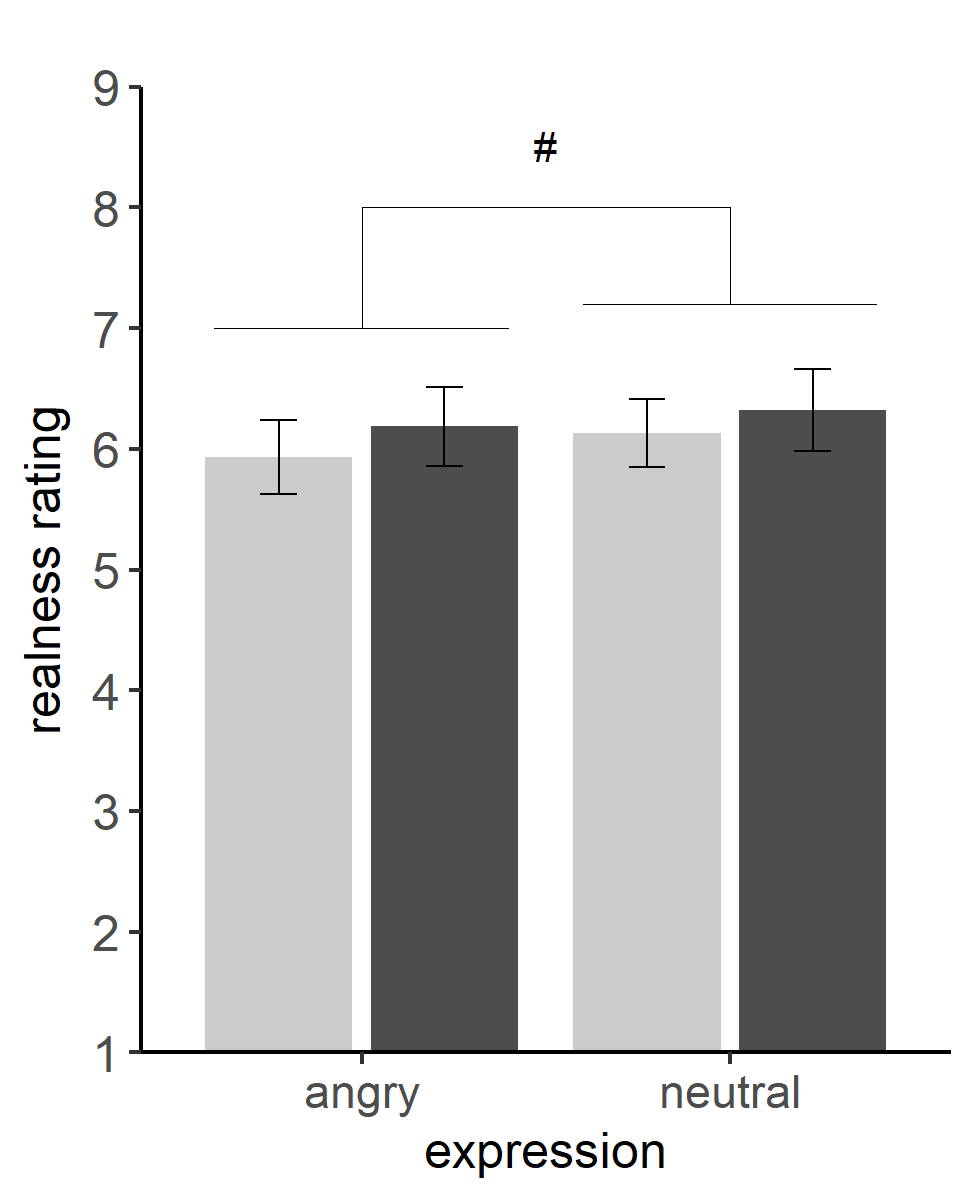

Supplement: S17 Fig — (# p < .1, * p < .05, ** p < .01, *** p < .001). (TIF) [file pone.0226805.s017.tif]
